# Evaluation of a Novel Hexavalent Humanized Anti-IGF-1R Antibody and Its Bivalent Parental IgG in Diverse Cancer Cell Lines

**DOI:** 10.1371/journal.pone.0044235

**Published:** 2012-08-31

**Authors:** Chien-Hsing Chang, Yang Wang, Preeti Trisal, Rongxiu Li, Diane L. Rossi, Anju Nair, Pankaj Gupta, Michele Losman, Thomas M. Cardillo, Edmund A. Rossi, David M. Goldenberg

**Affiliations:** 1 Immunomedics, Inc, Morris Plains, New Jersey, United States of America; 2 IBC Pharmaceuticals, Inc., Morris Plains, New Jersey, United States of America; 3 Center of Molecular Medicine and Immunology, Garden State Cancer Center, Morris Plains, New Jersey, United States of America; Vanderbilt University, United States of America

## Abstract

A major mechanism of monoclonal antibodies that selectively target the insulin-like growth factor type 1 receptor (IGF-1R) to inhibit tumor growth is by downregulating the receptor, regardless whether they are capable (antagonistic) or incapable (agonistic) of blocking the binding of cognate ligands. We have developed and characterized a novel agonistic anti-IGF-1R humanized antibody, hR1, and used the Dock-and-Lock (DNL) method to construct Hex-hR1, the first multivalent antibody comprising 6 functional Fabs of hR1, with the aim of enhancing potency of hR1. Based on cross-blocking experiments, hR1 recognizes a region of cysteine-rich domain on the α-subunit, different from the epitopes mapped for existing anti-IGF-1R antibodies, yet hR1 is similar to other anti-IGF-1R antibodies in downregulating IGF-1R and inhibiting proliferation, colony formation, or invasion of selected cancer cell lines in vitro, as well as suppressing growth of the RH-30 rhabdomyosarcoma xenograft in nude mice when combined with the mTOR inhibitor, rapamycin. Hex-hR1 and hR1 are generally comparable in their bioactivities under the in-intro and in-vivo conditions investigated. Nevertheless, in selective experiments involving a direct comparison of potency, Hex-hR1 demonstrated a stronger effect on inhibiting cell proliferation stimulated by IGF-1 and could effectively downregulate IGF-1R at a concentration as low as 20 pM.

## Introduction

Signals transmitted through cell surface growth factor receptors upon binding to cognate ligands are essential for regulating normal cell growth and differentiation, but also contribute to the development, proliferation, survival, motility, and metastasis of diverse types of malignant cells, as exemplified by the well-studied insulin-like growth factors (IGFs), and their main signaling receptor, IGF-1R [1]–[4]. The IGF signaling axis also consists of insulin as a ligand; three other homo-receptors, IGF-2R, insulin receptor isoform A (IRA), and insulin receptor isoform B (IRB); three hybrid-receptors, each formed from IGF-1R and IRA, IGF-1R and IRB, and IRA and IRB; six IGF binding proteins (IGFBRs); and a group of proteases that degrade IGFBPs to release IGFs. IGF-1R is a receptor tyrosine kinase, comprising two disulfide-linked extracellular α-subunits, each also disulfide-linked to a transmembrane β-subunit. The cytoplasmic region of the β-subunit harbors a tyrosine kinase domain, as well as a docking site for members of the insulin receptor substrate (IRS) family, and the SH2-containing adaptor protein, Shc [5]. IGF-1 binds to the α-subunits of IGF-1R with a higher affinity than IGF-2 [6]. The engagement of IGF-1R by IGFs induces autophosphorylation of the three tyrosine residues in the kinase domain of β-subunit [7], which further phosphorylates other tyrosine residues in the cytoplasmic domain, thereby leading to recruitment of IRS and Shc, with subsequent activation of both phosphoinositide 3-kinase (PI3K)-Akt and the mitogen-activated protein kinase (MAPK) pathways [8]. The minimal structural elements of the IGF-1 binding site on IGF-1R have been determined [9] to require the N-terminal L1 domain (aa 1–150), the C-terminus of the cysteine-rich domain (aa 190–300), and the C-terminus of the α-subunit (aa 692–702). In comparison, the functional epitopes of IGF-2 on IGF-1R were mapped [10] to involve the N-terminal L1 domain and the C-terminus of the α-subunit, but not the cysteine-rich domain. In addition to IGFBPs, the bioavailability of IGF-2 is also regulated by IGF-2R, which lacks intracellular kinase activity and thus functions as a scavenger receptor for IGF-2. Although IRB recognizes only insulin, its splice variant, IRA, which is most commonly expressed by tumors, also binds to IGF-2 [11] with high affinity, resulting in mitogenic effects and increased survival, motility, and invasiveness of cancer cells [12]. The complexity of the IGF-signaling system is further compounded by the ability of IGF-2 to stimulate IRA and IRA/IRB, the ability of both IGF-1 and IGF-2 to stimulate IGF-1R, IGF-1R/IRA, and IGF-1R/IRB, and the crosstalk between IGF-1R and EGFR [13]–[15], all of which appear to constitute pathways for certain cancer cells to escape IGF-1R-targeted therapies, and provide the rational for cotargeting IGF-1R with IR [16], [17] or EGFR/HER2 [18], [19] to enhance treatment efficacy.

The potential for targeting IGF-1R to treat cancers was demonstrated initially by the ability of αIR-3, a mouse monoclonal antibody (mAb) that blocks IGF-1R binding [20], to inhibit the in-vivo growth of the estrogen-independent MDA-MB-231 human breast cancer xenograft in nude mice [21]. Two main strategies for IGF-1R-targeted therapy (namely, blocking anti-IGF-1R antibodies and small molecule inhibitors of tyrosine kinase receptors) have been actively pursued over the past decade, resulting in various preclinical and clinical studies in diverse cancers, which were reviewed periodically [22]–[36]. In addition, the potential for dual inhibition of IGF-1 and IGF-2 with neutralizing antibodies was demonstrated more recently [37].

Most mAbs developed against IGF-1R to date were designed to spare IR and selectively inhibit IGF-IR-mediated signaling by blocking IGFs from binding. They also share a common property of inducing IGF-1R downregulation via internalization and degradation [38], which could inadvertently affect insulin signaling due to concurrent downregulation of the hybrid IGF-1R/IR receptors [39]. As summarized in Table S1 in the supplemental information *online*, these mAbs, besides being murine, humanized or fully human, differ in structural and functional properties that include isotype, epitope, potency to inhibit either or both IGFs, and affinity for IGF-1R. Eight such mAbs (AVE1642, BIIB022, cixutumumab, dalotuzumab, figitumumab, ganitumumab, R1507, and robatumumab) have been or are currently in clinical trials in various combinations. Whereas objective responses were reported from some phase II trials, for example, in two studies [40], [41] showing clinical benefit in non-small-cell-lung cancer (NSLCL) patients treated with figitumumab, paclitaxel and carboplatin (FPC), two subsequent phase III trials evaluating FPC and a combination of figitumumab with erlotinib in unselected patients with advanced NSCLC have been suspended in October 2009, owing to a lack of efficacy and a greater toxicity than anticipated from early-phase data [42]. Thus, despite a plethora of strong preclinical data supporting the rationale for IGF-1R-targeted cancer therapy, the generally disappointing clinical results, as revealed by figitumumab, R1507 (clinical development suspended in December 2009), and others [43], have pointed to a future direction that will focus on identification and validation of predictive biomarkers, as well as the investigation of more rational combinations with other anticancer agents to combat common resistance mechanisms developed from blockade of IGF-1R alone.

Multivalent antibodies often increase the potency of their bivalent parents, partly as a result of higher binding avidity to cognate antigens on target cells. Using the Dock-and-Lock (DNL) platform [44], [45] to generate a hexavalent antibody, designated Hex-hR1, from its bivalent parent, hR1, a novel humanized antibody (IgG1, *kappa*) that targets IGF-1R, but not IR, we explored the opportunity of comparing the various biological activities exhibited by Hex-hR1 and hR1 in diverse cancer cell lines, as well as their in vivo efficacy in a xenograft model of human rhabdomyosarcoma (RH-30) in nude mice, with or without the addition of rapamycin. We report herein that hR1 binds to a region of IGF-1R located in the mid-first half of the cysteine-rich domain (aa 185 −222), which is distinct from the epitopes reported for a number of anti-IGF-1R mAbs [46, 47, and Table S1]. The dissimilarity of hR1 to those anti-IGF-1R mAbs in clinical development also includes its inability to block the binding of either IGF-1 or IGF-2 to IGF-1R, and an intriguing behavior to induce phosphorylation of IGF-1R and downstream signaling without notably stimulating cell growth. On the other hand, hR1 is similar to other anti-IGF-1R antibodies in its ability to downregulate IGF-1R and inhibit proliferation, colony formation, or invasion of selected cell lines in vitro, as well as to retard the growth of the RH-30 xenograft in nude mice when combined with the mTOR inhibitor, rapamycin. Hex-hR1 and hR1 are generally comparable in their bioactivities under the conditions investigated, although Hex-hR1 did show a higher potency than hR1 in downregulating IGF-1R and in inhibiting proliferation of certain responsive cancer cell lines.

## Materials and Methods

### Cell Lines, Antibodies, and Reagents

All cell lines were purchased from ATCC, except RH-30, which was obtained from DSMZ. Humanized antibodies, including hR1, hPAM4 (anti-mucin), hA20 (anti-CD20), hRS7 (anti-Trop-2), hLL2 (anti-CD22), and hMN-15 (anti-CEACAM6), were provided by Immunomedics. Recombinant human IGF-1R (rhIGF-1R), recombinant human IGF-1, recombinant human IGF-2, and mouse anti-human IGF-1R mAb (MAB391) were obtained from R&D Systems. Additional anti-human IGF-1R antibodies were obtained from Calbiochem for Ab-1 (clone αIR3), Ab-3 (Clone 33255.111), and Ab-4 (clone 24–31); from Santa Cruz Biotechnology for 2C8, 1H7, 3B7, and C-20; from Millipore for 24–57 and 24–31; and from Invitrogen for 17–69, 24–60, and 1–2. Phospho-specific antibodies and other primary antibodies were acquired from Cell Signaling or Santa Cruz Biotechnology. Horseradish peroxidase (HRP)-conjugated secondary antibody and One Solution Cell Proliferation assay (MTS) were obtained from Promega. FITC- or TRITC-conjugated secondary antibodies were from Jackson ImmunoResearch Laboratories. PhosphoSafe Extraction Reagent and RIPA buffer used for cell lysis were obtained from EMD Biosciences and Cell Signaling, respectively. Cell culture media, supplements, and bovine transferrin (holo form) were bought from Invitrogen. Rapamycin (Sigma-Aldrich) was dissolved in DMSO, aliquoted, and stored at -20°C until use. Protein Assay Dye Reagent Concentrate was from Bio-Rad. All other chemicals were purchased from Sigma.

### Cell Culture

Malignant cell lines were routinely maintained at 37^o^ C in 5% CO_2_ in RPMI 1640 medium supplemented with 10% heat-inactivated fetal bovine serum (FBS), 1% GlutaMax I, 1% HEPES, 1% non-essential amino acids, and 1% sodium pyruvate (referred to as 10% RPMI). For Capan-1, 20% FBS was used (20% RPMI). The culture medium was changed at least once weekly and only cells with fewer than 50 passages were used for experiments.

### Generation of R1

Three BALB/c mice were each immunized i.p. with 15 µg of rhIGF-1R (Met1­Asn932), which comprises a mixture of both processed and unprocessed extracellular domain of human IGF-1R, in complete Freund’s adjuvant. Additional immunizations in incomplete Freund’s adjuvant were done 14, 21, and 28 days after the initial immunization. Hybridomas were generated by fusing the spleen cells from the immunized mice with P3×63Ag8.653 myeloma cells. One hybridoma clone (C-11), whose supernatant exhibited binding activity for IGF-1R but not IR, was isolated and expanded in cultures to obtain the mouse antibody designated R1 or mR1.

### Generation of cR1

Chimerization of R1 to obtain cR1 was performed as follows. The V_H_ and V_K_ genes of R1 were cloned by 5′-RACE. The DNA sequences of the cloned V_H_ and V_K_ genes were determined with the authenticity confirmed by N-terminal protein sequencing that showed an exact match of the first 15 N-terminal amino acids with the corresponding amino acids deduced from DNA sequences (Table S2). The cloned V_H_ and V_K_ genes were inserted into the *pdHL2* vector to generate *cR1pdHL2*, which was used to transfect SpE-26 cells, a variant ofSp2/0-Ag14 developed in-house. Transfectants were selected with 0.075 µM methotrexate (MTX), and screened by ELISA for human Fc binding activities. Higher producing clones were further expanded to obtain the two best clones (709.2D2 and 710.2G2), from which cR1 was produced in batch cultures and purified by Protein A chromatography.

### Generation of hR1

Humanization [48] of cR1 to hR1 was achieved by grafting the CDRs onto the human framework regions of hMN-14. For certain framework positions, murine residues of R1 were retained, resulting in the amino acid sequences of hR1 V_H_ (Figure S1A) and hR1 V_K_ (Figure S1B). Synthetic genes encoding hR1 V_H_ and hR1 Vk were engineered into *pdHL2* to obtain *hR1pdHL2*, the expression vector for hR1. Subsequent efforts to secure the production clone (711.3C11) for hR1 were similar to those described above for cR1, except that the positive clones were selected for binding activities to both human Fab and rhIGF-1R.

### Generation of Hex-hR1 by DNL

C_H_1-DDD2-Fab-hR1 and C_H_3-AD2-IgG-hR1 were produced as fusion proteins in SpESF cells [49] transfected with the respective vectors as described for C_H_1-DDD2-Fab-hA20 and C_H_3-AD2-IgG-hA20 [50]. Hex-hR1 was obtained as follows. C_H_1-DDD2-Fab-hR1 was mixed with C_H_3-AD2-IgG-hR1 in phosphate buffered saline (PBS), pH 7.4, with 1 mM EDTA, at a molar ratio of 4.2 to effect the conjugation of most, if not all, C_H_3-AD2-IgG-hR1 to C_H_1-DDD2-Fab-hR1, thus reducing the potential co-purification of C_H_3-AD2-IgG-hR1 with the final product upon protein A column chromatography. The DNL reaction was initiated by adding reduced glutathione (GSH) to 1 mM, followed by adding oxidized glutathione (GSSH) to 2 mM on the next day, and after a further incubation overnight, Hex-hR1 was purified from the resulting solution by protein A chromatography.

### Purity, Size, and Mass Analyses

Size-exclusion high performance liquid chromatography (SE-HPLC) was performed on a Beckman System Gold Model 116 with a BioSep-SEC-s3000 column (300×7.80 mm) of Phenomenex using 0.04 M PBS (pH 6.8) plus 1 mM EDTA as the mobile phase to determine the molecular integrity and product purity of mAbs and HexAbs. The average hydrodynamic diameters of hR1 and Hex-hR1 were determined by dynamic light scattering (DLS) with a contract to Microtrac. Electrospray ionization time of flight (ESI-TOF) liquid chromatography/mass spectrometry (LC-MS) was performed on a 1200-series HPLC coupled with a 6210 TOF MS (Agilent Technologies). Briefly, hR1 or Hex-hR1 was reduced with 50 mM tris(2-carboxyethyl)phosphine for 30 min and resolved by reversed phase HPLC (RP-HPLC), using a 20-min gradient of 30–80% acetonitrile in 0.1% aqueous formic acid with a Jupiter C4 5µ column (Phenomenex). For the TOF MS, the capillary and fragmentor voltages were set to 5500 and 250 V, respectively.

### Competition Binding Studies

Homogeneous polystyrene microsphere beads coated with rhIGF-1R to serve as surrogates of cells expressing IGF-1R were used in all competition binding studies. To compare the binding affinity, varying concentrations of unlabeled mR1, cR1, and hR1 were mixed with a constant amount of Alexa Fluor 532-labeled cR1 (532-cR1). The coated beads were added to a final density of 2×10^5^ particles/mL and the mixtures were incubated at room temperature (RT) for 1 h with gentle rocking. The bound 532-cR1 was determined by measuring median fluorescence intensity (MFI) of 2,000 beads on a Guava PCA System (Millipore).

To determine whether cR1 can block the binding of IGF-1 or IGF-2 to IGF-1R, varying concentrations (0 to 670 nM) of cR1, IGF-1, or IGF-2 were mixed with a constant amount of ^125^I-IGF-1 or ^125^I-IGF-2. The coated beads were then added, incubated at RT for 1 h with gentle rocking, washed, and counted for radioactivity.

To probe the binding region of hR1 on IGF-1R, a panel of commercially available anti-IGF-1R mAbs, with their epitopes to IGF-1R mapped (except MAB391), were evaluated as competitors for blocking hR1 from binding to the rhIGF-1R-coated beads. In these experiments, R1, cR1, hR1, MAB391, 24–60, and αIR-3 were each labeled with R-phycoerythrin (PE) and incubated with an unlabeled antibody of interest at varying concentrations.

### Binding to Cell Surface IGF-1R

Each sample was prepared in duplicate to contain 2×10^5^ cells and 10 µg/mL of a test antibody in a final volume of 200 µL. After incubation at 4°C for 45 min, samples were washed twice with PBS-1% BSA, followed by the addition of FITC-GAH IgG, (H + L), and a further incubation at 4°C for 45 min in the dark. Samples were then washed twice with PBS-1% BSA, resuspended in 500 µL of PBS-buffered formalin, and analyzed on FACScan.

### Cell Proliferation Assay

All incubations of cells were performed at 37°C in a humidified 5% CO_2_ incubator. Cells were detached with trypsin, washed three times with PBS to remove any trace of serum, and resuspended in a serum-free medium containing 10 µg/mL of bovine transferrin (SFM-Trf). Cells were seeded at 1.0×10^3^ cells/50 µL/well and incubated overnight. On the following day each test article in SFM-Trf was 5-fold serially diluted from 400 nM to 0.001 nM and 50 µL of each concentration were added in triplicate to the wells such that the final concentrations of the test article ranged from 200 nM to 0.0005 nM. Untreated control cells received only 50 µL of SFM-Trf. After incubation for 1 h, designated wells received 100 µL of each test article at the same concentration in SFM-Trf containing 50 ng/mL of IGF-1. Plates were then incubated for a period of time as indicated and the number of viable cells in each well was determined using the MTS assay per the manufacturer’s protocol.

### Colony Formation Assay of Cells Grown in Monolayer Culture

DU 145 Cells were detached with trypsin and plated in 60-mm dishes (1×10^3^ cells) in 10% RPMI supplemented with 1% penicillin/streptomycin (P/S). Test articles were added and medium containing the test articles was replaced every four days, After 14 days, cells were fixed in 4% para-formaldehyde and stained with 5% Giemsa solution. Colonies greater than 50 cells were enumerated under a microscope. In a separate experiment, test articles were not added to the subsequent medium.

### Colony Formation Assay of Cells Grown in Soft Agar

Basal agar (0.5%) was prepared by mixing 1% agar (at 40°C) with an equal volume of 2×10% RPMI and added to each well (0.5 mL) in a 24-well plate. Cells in 2×10% RPMI were mixed with an equal volume of 0.7% agarose and added (0.5 mL) to the top of the base agar for the final cell count of 1250 per well in 0.35% agarose. Cells were fed with 0.5 mL of 10% RPMI added to each well weekly. Treated wells contained the test articles in the agarose/cell layer at the beginning and in subsequent feedings. Once colonies were clearly visible by microscopy in untreated control wells, the medium was removed and the colonies stained with crystal violet. Colonies were counted under a microscope and the average number was determined from five different fields of view within the well.

### Immunoblot Analysis

Unless otherwise stated, cells were starved in serum-free medium for 24 h, treated, and lysed at ice-cold temperature in a buffer as specified. Protein concentrations were determined by the Bio-Rad Protein Assay and samples (15 to 30 µg loaded in each lane) were separated on 4–20% Tris-Glycine gels, transferred to PDVF or nitrocellullose membranes, blocked with TBST buffer (50 mM Tris pH 8.0, 150 mM NaCl, 0.1% Tween 20) containing 5% nonfat milk, washed with TBST buffer, and incubated overnight at 4°C with primary antibodies. The membranes were then washed in TBST four times (once for 15 min and three more for 5 min each), incubated with HRP-conjugated secondary antibodies for 1 h at RT, washed in TBST buffer four times as described above, then detected with Super Signal West Dura Extended Duration Substrate (Thermo Scientific) according to the directions provided by the manufacturer. The immunoblot signals were visualized with a chemiluminescence system (Thermo Scientific). Digital images were processed by Carestream (Carestream Molecular Imaging).

### Downregulation of IGF-IR

Cells in 10% RPMI were seeded at 1×10^6^ per 100-mm dish and cultured overnight for attachment. On the next day, the medium was replaced with fresh 10% RPMI containing a test article of interest at indicated concentrations and cells were further incubated for 24 h or a predetermined time. For analysis by Western blot, treated cells were washed with cold PBS, scraped from the dishes, collected, and centrifuged at 4°C at 2,000 rpm for 5 min. Cells pellets were lysed for 10 min on ice in RIPA buffer or a buffer consisting of 25 mM Tris (pH 8), 150 mM NaCl, 1 mM EDTA, 1% Triton and 1× Complete, EDTA-free Protease Inhibitor Cocktail (Roche Diagnostics). The lysates were clarified by centrifugation, assayed for protein concentration, and analyzed by immunoblotting. For analysis by flow cytometry, treated cells were washed in PBS twice, incubated with PE-labeled 1H7 for 1 h, washed twice with PBS, resuspended in 500 µL of PBS, and analyzed on FACScan.

### Phosphorylation of IGF-IR and Akt

Cells (5×10^5^ per well) were grown in 10% RPMI in 6-well plates overnight for attachment. Following two washes with serum-free medium, cells were incubated for 4 h in serum-free medium and treated with test articles at indicated concentrations for a specified time. Cells were then stimulated with IGF-I for 10 min, washed with PBS, lysed with 200 µL of RIPA buffer for 5 min at RT, and processed for immunoblot analysis.

### Matrigel Invasion Assay

The manufacturer’s protocol on BD BioCoat™ Matrigel™ Invasion Chamber (BD Biosciences) was followed, using the 24-well format, which provides 12 inserts, each containing an 8-µm pore size membrane with a thin layer of MATRIGEL Basement Membrane Matrix. Before use, the 24-well assembly was removed from storage at −20°C and allowed to warm to RT. The interior of the inserts and the bottom of the wells were rehydrated with warm (37°C) bicarbonate-based culture medium for 2 h, and carefully removed. Cells (0.5 mL at 5×10^4^/mL) in serum-free medium were placed in the insert (upper chamber) and the well (lower chamber) was filled with 0.5 mL of 10% RPMI. After 2 h, test articles were added to cells in the upper chamber and the incubation continued for 20 h, at which time cells that remained in the Matrigel or attached to the upper side of the membrane were removed with cotton tips. Cells on the lower side of the membrane were fixed in methanol, stained with either Wright-Giemsa stain or Hoechst 33258, and examined under a fluorescent microscope.

### Immunofluorescence Microscopy

Cells grown on coverslips were washed, fixed with 4% formalin, washed, incubated with primary antibodies for 1 h at RT, washed with PBS, and reacted with FITC- or TRITC-conjugated secondary antibodies at RT for 40 min. After washing, the samples were stained with Hoechst 33258, mounted, and examined under a fluorescent microscope.

### In vivo Efficacy

Female 8-week-old SCID mice (Taconic Farms) were used. Each mouse was injected s.c. with 5×10^6^ RH-30 cells. Once tumors reached approximately 0.2 cm^3^ in size, the animals were divided into six groups of 10 mice each and injected i.p. twice weekly for four weeks with hR1 (1 mg), Hex-hR1 (0.82 mg), rapamycin (5 mg/kg), hR1 (1 mg) plus rapamycin (5 mg/kg), Hex-hR1 (0.82 mg) plus rapamycin (5 mg/kg), and saline (containing 2% DMSO), respectively. A stock solution of rapamycin was prepared in saline (containing 2% DMSO) at 1 mg/mL and 100 µL were administered to each mouse per injection. Tumors were measured and mice weighed twice weekly. Animals were sacrificed when tumors reached 2 cm^3^. A second study to compare the efficacy of hR1 and Hex-hR1 given at molar equivalent doses (0.33 mg vs. 0.82 mg) also was performed. Protocols for animal studies were approved by the CMMI Institutional Animal Care and Use Committee.

### Statistical Analysis

For in vitro studies, the statistical difference between two populations was determined by Student’s *t*-test. Statistical analysis for the tumor growth data was based on area under the curve (AUC) and median survival time using a two-tailed *t*-test to assess significance between all the various treatment groups and controls, except the saline control, for which a one-tailed *t*-test was used. Survival curves were analyzed by Kaplan-Meier plots (log-rank analysis), using the Prism GraphPad software package (v4.03; Advanced Graphics Software, Inc.). A value of *P*<0.05 was considered statistically significant.

## Results

### Notable Properties of R1, cR1 and hR1

The parental R1 was shown to partially inhibit the binding of ^125^I-labeled IGF-1 (^125^I-IGF-1) to the human breast cancer cell line MCF-7L (a subline of MCF7) comparable to MAB391 (Table S3). Chimerization of R1 appeared to improve the affinity of R1 for rhIGF-1R immobilized onto polystyrene beads, as shown by a competition assay in which the binding of R1 tagged with a fluorescent probe (Alexa 532) was measured by flow cytometry in the presence of varying concentrations of cR1 or R1 (Figure S2). Antibodies produced by the two clones of cR1 showed the same affinity of 0.1 nM (Figure S3) and were specific for immobilized rhIGF-1R but not immobilized rhIR (Figure S4). However, cR1 failed to block the binding of IGF-1 or IGF-2 to immobilized rhIGF-1R in the bead assay (Figure S5), contrary to the earlier observation that its murine counterpart could partially inhibit the binding of ^125^I-IGF-1 to MCF-7L cells. Successful humanization was demonstrated by the equivalent potency of hR1 and cR1 to compete with 532-cR1 for binding to rhIGF-1R-immobilized beads (Figure S6). On the other hand, the bead assay also showed hR1 was ineffective in inhibiting the binding of ^125^I-IGF-1 to the immobilized rhIGF-1R (Figure S7). Based on the results from the cross-blocking experiments (Tables 1 and 2), the epitope of hR1 was deduced to reside between the amino acid residues 185 and 222 in the mid-first half of the cysteine-rich domain. Intriguingly, although MAB391 had no effect on the binding of PE-labeled R1 to immobilized rhIGF-1R (Figure S8A), R1 substantially reduced the binding of PE-labeled MAB391 (Figure S8B), suggesting that R1 may inhibit the binding of MAB391 to immobilized rhIGF-1R allosterically.

**Table 1:** Epitope analysis by cross-blocking with anti-IGF-1R antibodies of known epitopes.

	Epitope	3B7	24–31	2C8	24–57	1H7	17–69	1–2
Epitope		62–184	283–440	301–450	440–514	440–514	514–586	1323–1337
mR1*	(185–282)	117	100	100	100	150	100	100
cR1*	(185–282)	106	100	100	100	125	100	100
hR1*	(185–282)	100	100	100	100	131	100	100
MAB391*	?	ND	ND	ND	ND	ND	ND	ND
24–60*	184–283	79	18	88	88	100	82	100
αIR-3*	223–274	76	52	97	87	115	89	95

The values shown are percent (%) binding of each PE-labeled antibody (*) to the extracellular domain of rhIGF-1R (M1-M932) in the presence of an unlabeled antibody at the highest testing concentration of 100 µg/mL. These data indicate that the epitope of hR1 is likely located between the amino acid residues 185 and 282.

**Table 2:** Epitope analysis by cross-blocking with anti-IGF-1R antibodies of known epitopes (continued).

	mR1	cR1	hR1	24–60	αIR-3	MAB391
Epitope	(185–282)	(185–282)	(185–282)	184–283	223–274	?
			185–222			
mR1*	0	0	0	43	143	100
cR1*	0	0	0	40	128	108
hR1*	0	0	0	71	136	121
24–60*	0	0	0	0	21	0
αIR-3*	86	97	107	0	0	0
MAB391*	40	ND	ND	ND	ND	0

These data further narrow the epitope of hR1 to be between the amino acid residues 185 and 222 located in the mid-first half of the cysteine-rich domain. Note that mR1, cR1, and hR1 crossblock 24–60 and may allosterically inhibit the binding of MAB391.

### Molecular Characterization of hR1 and Hex-hR1

**Figure 1:**
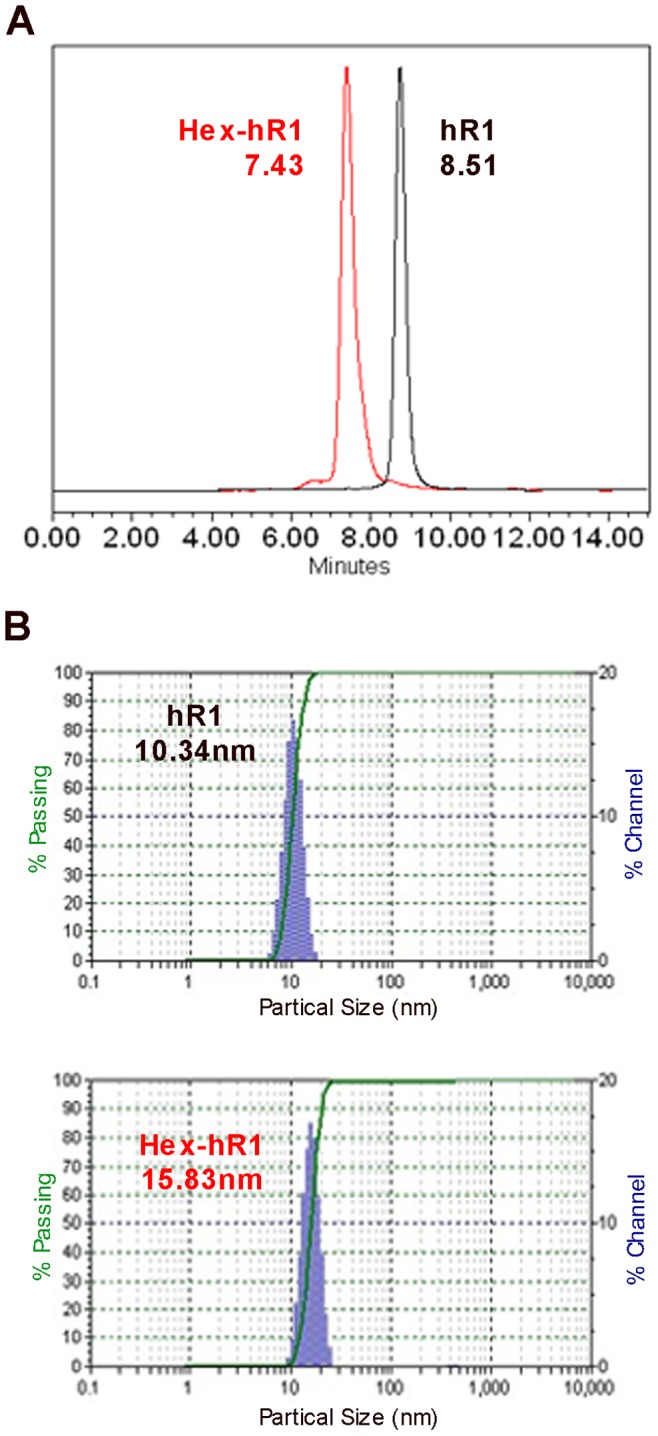
Molecular purity and size of hR1 and Hex-hR1 shown by SE-HPLC (A) and DLS (B).

The SE-HPLC and DLS profiles of hR1 and Hex-hR1, shown in Fig. 1A and 1B, respectively, indicate their high degree of homogeneity. For hR1, a single peak at 8.51 min was observed. Hex-hR1, which comprises a pair of stabilized dimers of hR1 Fab appended to a full hR1 IgG at the carboxyl termini of the two heavy chains, also displayed only a major peak at 7.43 min. The particle sizes of hR1 and Hex-hR1 were largely monodisperse, with the average hydrodynamic diameters of 10.34 nm and 15.83 nm, respectively.

As shown in Table 3, the mass of each of the polypeptides comprising hR1 and Hex-hR1 determined by LC/MS was consistent with the mass calculated from their deduced amino acid sequence and predicted post-translational modifications, including N-linked glycosylation and amino-terminal pyroglutamate on the heavy chain of hR1 and Hex-hR1, and the Fd-DDD2 chain of Hex-hR1. For both hR1 and Hex-hR1, the *kappa* chain, which is not altered, gave a nearly identical mass observed within 20 ppm of the predicted mass. For hR1, the heavy chain was detected as several isoforms, including those of carboxyl-terminal lysine variants and various glycoforms. For Hex-hR1, the AD2-fused heavy chain was intact, with predominantly G0F and G1F glycoforms. The the hR1-Fd-DDD2 component of Hex-hR1 also matched that of the predicted mass within 0.1 ppm, with no additional post-translational modifications besides the amino-terminal pyroglutamate.

**Table 3:** LC-ESI-TOF-MS analysis of hR1 and Hex-hR1.

Ab	Polypeptide	Modifications	M_calc_	M_obs_	Diff (mDa)	Diff (ppm)
hR1	Kappa	None	23364.2082	23363.7853	−422	−18.1
	Heavy Chain	pQ; ΔK; G0F	51086.6403	51087.1519	511	10
	Heavy Chain	pQ; G0F	51214.8153	51216.9697	2154	42
	Heavy Chain	pQ; ΔK; G1F	51248.7836	51249.5685	785	15
	Heavy Chain	pQ; G1F	51376.9586	51378.8727	1914	37
	Heavy Chain	pQ; ΔK; G2F	51410.9268	51411.2145	288	6
	Heavy Chain	pQ; G2F	51539.1018	51540.5513	1449	28
Hex-hR1	Kappa	None	23364.2082	23364.3416	133	6
	Fd-DDD2	pQ	29889.6339	29889.6366	3	0.1
	Heavy chain-AD2	pQ; G0F	54035.9341	54036.3958	462	9
	Heavy chain-AD2	pQ; G1F	54198.0774	54198.6210	544	10


pQ, N-terminal pyroglutamate; ΔK, loss of C-terminal lysine; G0F, G1F and G2F, N-linked glycans; M_calc_, calculated mass; M_obs_, observed LC/MS mass, mDa, millidaltons; ppm, parts per million.

### Expression of IGF-1R in Diverse Cancer Cell Lines

Positive binding by hR1 and Hex-hR1 was demonstrated in MCF7 (breast cancer), DU 145 (prostate cancer), ME-180 (cervical cancer) and RH-30 (rhabdomyosarcoma) by flow cytometry (Table 4). Based on the observed median fluorescence intensity (MFI), the expression levels of IGF-1R varied among these four cell lines, with the relative abundance of the receptor in RH-30 increased nearly 2-fold in DU 145 or ME-180, and about 3-fold in MCF7. In light of the number of surface IGF-1Rs per cell as reported for RH-30 [51] and MCF7 [52] to be 20,600 and 43,000, respectively, there would be a 2.3-fold increase in MCF7 when compared with RH-30, which agrees well with the 3-fold increase estimated from the MFI data. Additional studies with hR1 have found overexpression of IGF-1R, at a level comparable with that of RH-30, in other cancer cell lines, which include Capan-1 and BxPC-3 of pancreatic cancer, LNCaP of prostate cancer, Colo205 and HT-29 of colon cancer, HepG2 and Huh-7 of liver cancer, ACHN and 786-O of kidney cancer, SK-OV-4 of ovarian cancer, A375 of melanoma, A549 and SK-MES-1 of lung cancer, KMS11 of multiple myeloma, and RD of rhabdomyosarcoma (data not shown).

**Table 4:** IGF-1R expression as determined by flow cytometry.

	MFI (% positive)			
	MCF7	DU 145	ME-180	RH-30
FITC-GAH	3.84 (0.89)	6.22 (4.24)	3.11 (3.36)	3.29 (2.81)
+ hA20	3.85 (0.83)	5.58 (3.57)	3.15 (3.45)	3.48 (3.25)
+hR1	79.22 (99.87)	50.62 (97.69)	41.56 (99.63)	26.30 (99.26)
+ Hex-hR1	55.91 (99.78)	42.78 (96.44)	44.64 (99.60)	21.44 (97.44)

### Effects of hR1 and Hex-hR1 on Cell Proliferation in Serum-free Medium

The rationale for selecting MCF7, RH-30, DU 145, and ME-180 as representative cell lines to study the potential activity of hR1 and Hex-hR1 in affecting cancer cell proliferation was based on the knowledge that they all express high levels of IGF-1R, as assessed by hR1, and their responses to other anti-IGF-1R antibodies, for example, MCF7 and DU 145 to EM164 [52], and RH-30 to h7C10 [51], were described in the literature. It is noted that the in vitro sensitivity of a certain cancer cell line to growth inhibition by an anti-IGF-1R mAb has been proposed to require a minimal level of IGF-1R expression as well as a functional IGF signaling axis, both of which may contribute to ligand-stimulated proliferation [53]. Other studies have correlated high expression levels of IGF-1R with sensitivity to anti-IGF-1R antibodies [51], [54].

**Figure 2:**
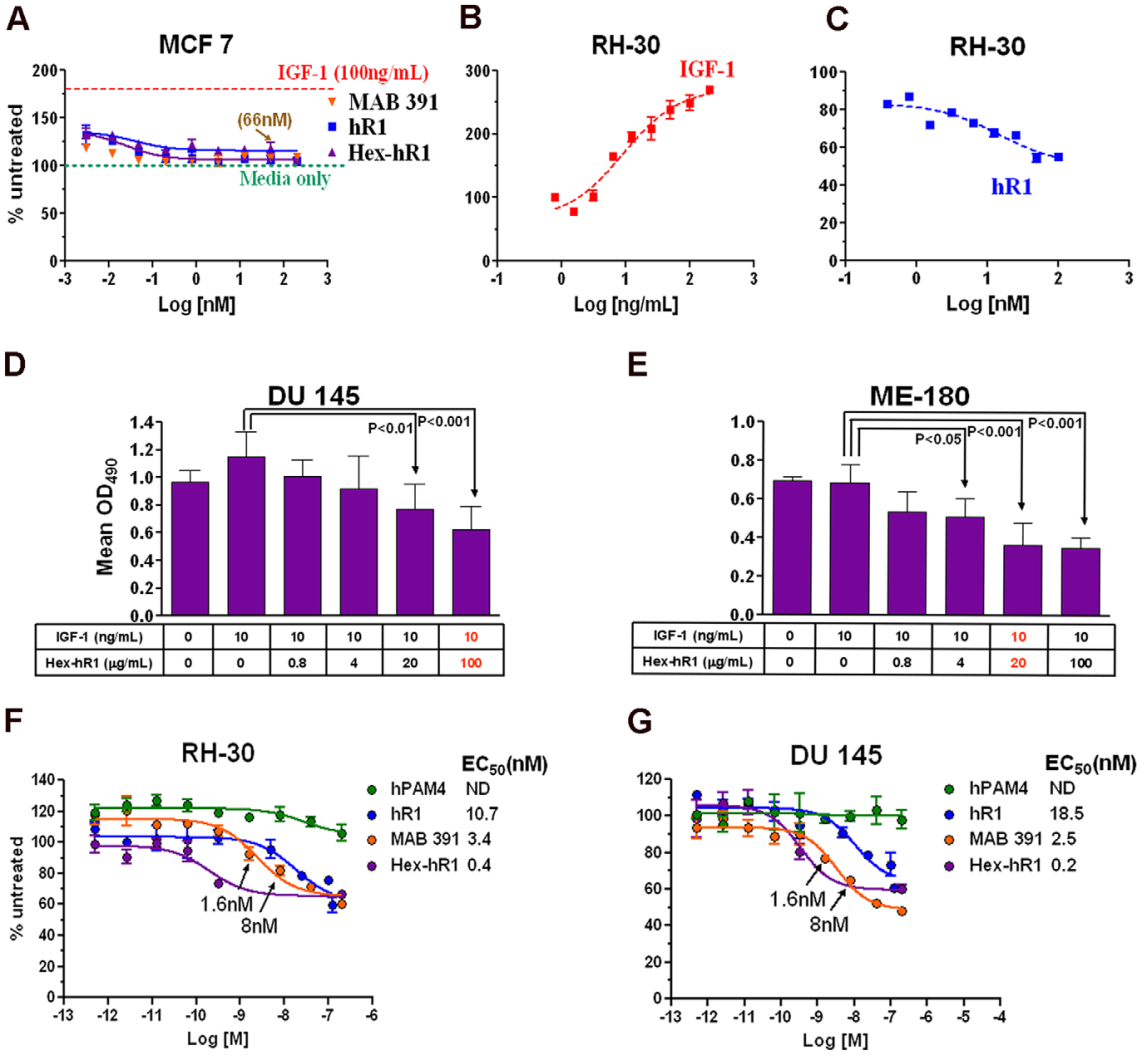
Various cell proliferation studies in serum-free medium as determined by the MTS assay. (A) Lack of growth promotion by MAB391, hR1 or Hex-hR1 on MCF7; (B) Dose-response stimulation of RH-30 proliferation by IGF-1; (C) Sensitivity of RH-30 to hR1; (D) Inhibitory activity of Hex-hR1 observed in DU 145 stimulated with 10 ng/mL of IGF-1 for 72 h; (E) Inhibitory activity of Hex-hR1 observed in ME-180 stimulated with 10 ng/mL of IGF-1 for 72 h; (F) Dose-dependent inhibitory effects of hR1 and Hex-hR1 on proliferation of RH-30 in the presence of 50 ng/mL of IGF-1. MAB391 and hPAM4 were tested in parallel as positive and negative controls, respectively; (G) Dose-dependent inhibitory effects of hR1 and Hex-hR1 on proliferation of DU 145 in the presence of 50 ng/mL of IGF-1. In A, B, C, F and G, the number of viable cells determined for each treated sample was expressed as percent (%) of the untreated control. EC_50_ values were calculated and indicated in (F) and (G).

As shown in Fig. 2A, the proliferation of MCF7 cells in SFM-Trf was increased to about 175% in the presence of 100 ng/mL of IGF-1, whereas the addition of hR1, Hex-hR1 or MAB391 up to 200 nM had no stimulatory or inhibitory effect. Under the same culture conditions, the proliferation of RH-30 cells, which were more responsive to IGF-1, achieving a growth enhancement of 250% at 100 ng/mL (Fig. 2B), could be moderately inhibited by hR1 (up to 40% at 100 nM), as shown in Fig. 2C. In a 72-h assay, the effect of IGF-1 (10 ng/mL) on the proliferation of DU 145 cells (Fig. 2D) was reduced by Hex-hR1 about 30% at 20 µg/mL (*P*<0.01) and about 50% at 100 µg/mL (*P*<0.001). In a similar study with ME-180 cells, which were insensitive to IGF-1, a 50% reduction in proliferation (Fig. 2E) could be achieved with Hex-hR1 at 20 µg/mL (*P*<0.001) in the presence of 10 ng/mL of IGF-1. Additional studies indicated that Hex-hR1 was more potent than either hR1 or MAB391 in inhibiting the proliferation of RH-30 or DU 145 in SFM-Trf containing 50 ng/mL of IGF-1. Specifically, a maximal reduction of 40% in cell proliferation of RH-30 could be attained with Hex-hR1 at 1.6 nM (Fig. 2F), which was 25-fold lower than hR1. A similar potency profile was observed in DU 145 (Fig. 2G). These results are further presented as EC_50_ values to highlight the enhanced potency of Hex-hR1 in comparison to hR1 or MAB391.

### Effects of hR1 and Hex-hR1 on Colony Formation in Monolayer Culture

**Figure 3:**
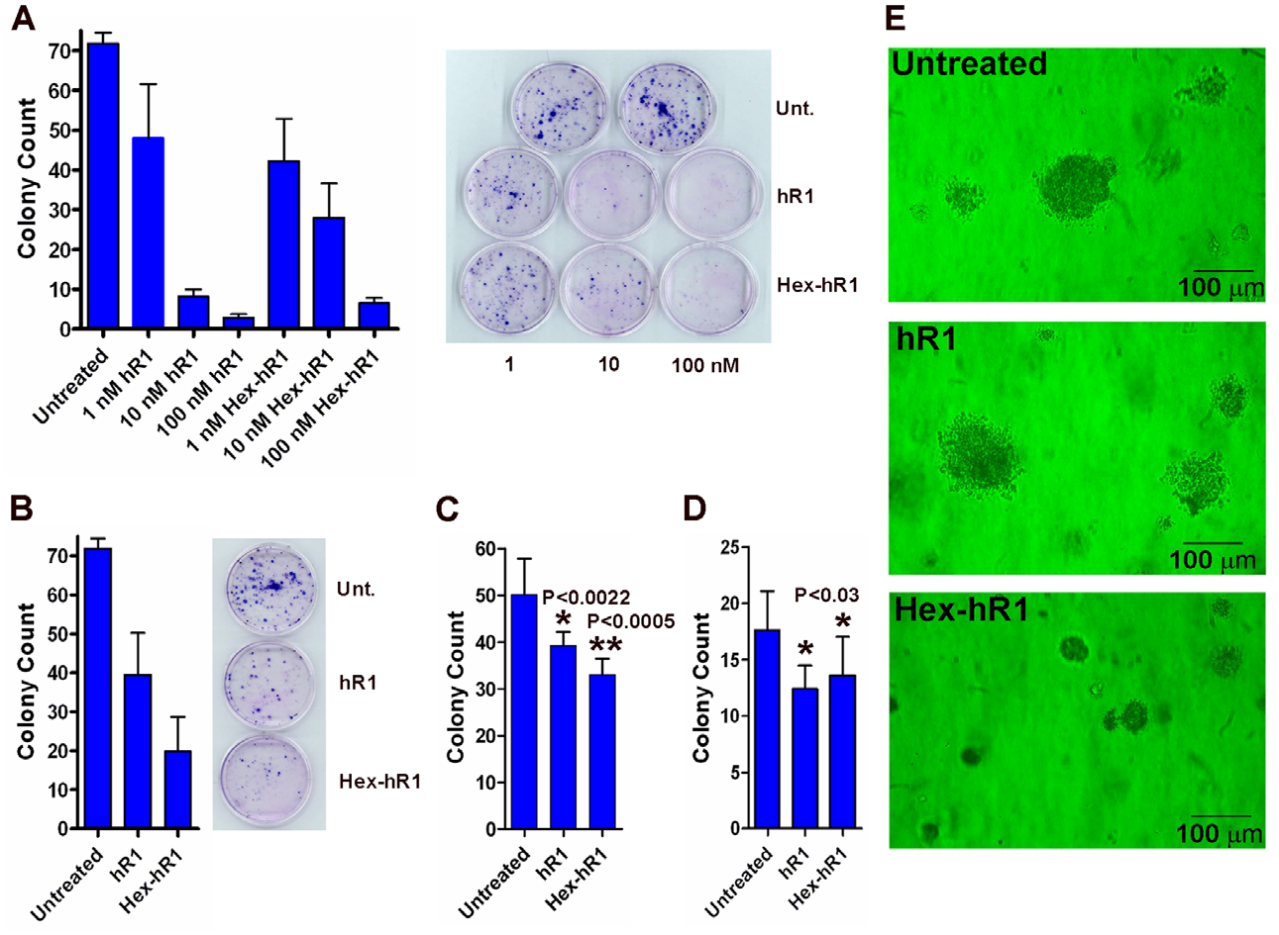
Inhibition of colony formation of DU 145 in monolayer culture with continuous (A) or single exposure (B) to hR1 or Hex-hR1 at 1, 10, and 100 nM; **inhibition of anchorage-independent growth of ACHN (C) and 786-O (D) by hR1 and Hex-hR1 in soft agar; (E) representative images of the colonies formed by cells treated or not with hR1 or Hex-hR1.**

The potential of hR1 or Hex-hR1 to affect cell growth in monolayer culture was evaluated in DU 145 cells using a clonogenic assay. Fig. 3A shows that both hR1 and Hex-hR1 were effective in inhibiting colony formation of DU 145 in 10% RPMI at the three test concentrations of 1 (*P*<0.04 vs. untreated), 10 (*P*<0.01 vs. untreated), and 100 nM (*P*<0.01 vs. untreated). In a separate study in which cells were treated only once with hR1 or Hex-hR1 (since media of subsequent changes were added without the antibodies), colony formation was also significantly reduced (Fig. 3B) by 100 nM of hR1 (*P*<0.01 vs. untreated) or Hex-hR1 (*P*<0.01 vs. untreated), with Hex-hR1 more potent than hR1 (*P*<0.031).

### Effects of hR1 and Hex-hR1 on Anchorage-independent Colony Formation

The soft agar assay was performed initially in MCF7 and MDA-MB-231, with the results depicting that hR1 at 200 nM had no effect on colony formation of either cell line (data not shown). Subsequent studies in two renal carcinoma lines, however, demonstrated that both hR1 and Hex-hR1 at 200 nM significantly reduced the number of colonies formed by ACHN (Fig. 3C) and 786-O (Fig. 3D) when compared to an untreated control (*P*<0.03). The colonies of cells treated with Hex-hR1 were considerably smaller in size, as shown in Fig. 3E.

### Downregulation of IGF-1R

**Figure 4:**
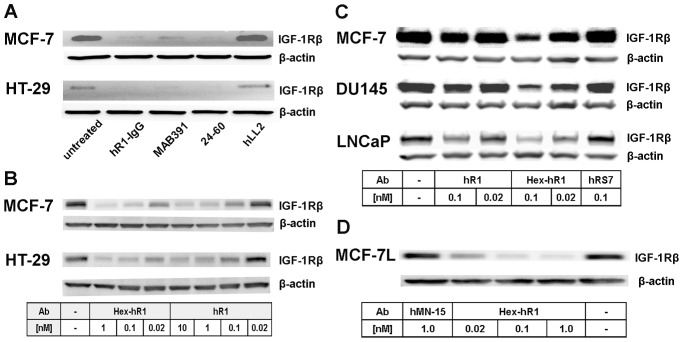
Downregulation of IGF-1R revealed with Western blot analysis of IGF-1Rβ in (A) MCF7 and HT-29 cells treated with hR1, MAB391, 24–60, and hLL2; **(B) MCF7 and HT-29 cells treated with hR1 or Hex-hR1; (C) MCF7, DU 145, and LNCaP cells treated with hR1, Hex-hR1, or hRS7 (irrelevant IgG); (D) MCF-7L cells treated with Hex-hR1 or hMN-15 (irrelevant IgG).** In all experiments, β-Actin was used as a loading control.

One major mechanism of anti-tumor action induced by an anti-IGF-1R antibody, regardless of its being an agonist or antagonist, is to downregulate IGF-1R [55]. Efficient downregulation of IGF-1R in MCF7 or HT-29 was demonstrated initially with hR1 at 100 nM (Fig. 4A). Follow-up studies revealed that Hex-hR1 at 0.02 nM and hR1 at 0.1 nM resulted in an appreciable reduction of IGF-1R in HT-29 (Fig. 4B), MCF7 (Fig. 4C and Fig. 4D), DU 145 (Fig. 4D), and LNCaP (Fig. 4D). Densitometry analysis of the Western blots (Fig. 4E) showed a more than 50% decrease in the band intensity of IGF-1R prepared from MCF-7L cells treated with 0.02 M Hex-hR1. In cells treated with the irrelevant hRS7 (Fig. 4D) or hMN-15 (Fig. 4E), the level of IGF-1R was not affected. Effective downregulation of IGF-1R in MCF7 and DU 145 following treatment with either hR1 or Hex-hR1 at 10 nM overnight was also demonstrated by flow cytometry, as shown in Figure S9.

### Phosphorylation of IGF-1R and Akt

**Figure 5:**
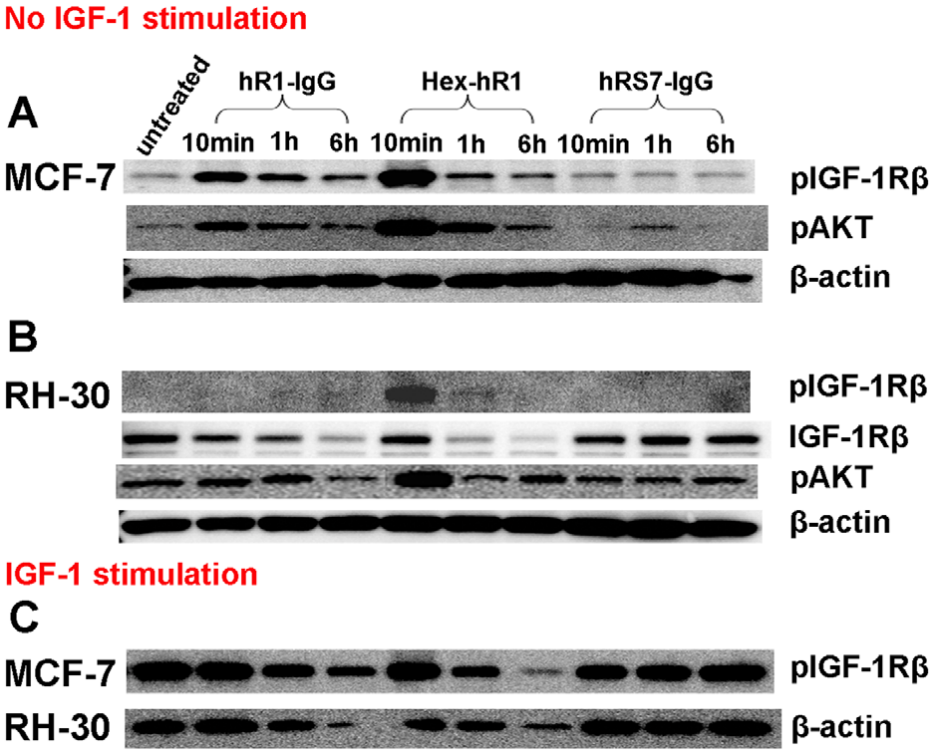
Phosphorylation of IGF-1R and Akt. (A) hR1, Hex-hR1, but not hRS7 (irrelevant IgG) induced phosphorylation of IGF-1Rβ and Akt in MCF7 cells in the absence of IGF-1; (B) under the same conditions as in (A), only Hex-hR1 was effective in inducing phosphorylation of IGF-1R1Rβ and Akt in RH-30 cells; (C) MCF7 and RH-30 cells were treated as in (A) except with the addition of IGF-1.

An anti-IGF-1R antibody is considered to be an agonist if its binding to IGF-1R leads to autophosphorylation of the receptor. As shown in Fig. 5A, both hR1 and Hex-hR1 at a concentration of about 70 nM effectively induced phosphorylation of IGF-1R in MCF7 cells when analyzed at 10 min, 1 h and 6 h, which subsided with time and was accompanied by a parallel change in phosphorylated Akt. Similar changes in the phosphorylation of IGF-1R and Akt were observed in RH-30 (Fig. 5B) for Hex-hR1, but not hR1. The decrease in the levels of phosphorylated IGF-1R was attributed to the downregluation of IGF-1R as revealed by the finding that in MCF7 and RH-30 cells stimulated by IGF-1 after a similar treatment with hRS7 (a non-anti-IGF-1R antibody), the levels of phosphorylated IGF-1R induced at all three time points were the same as that of the untreated cells (Fig. 5C, lanes 8–10 vs. lane 1).

### Effects on Cell Invasion, E-cadherin, and Vimentin

**Figure 6:**
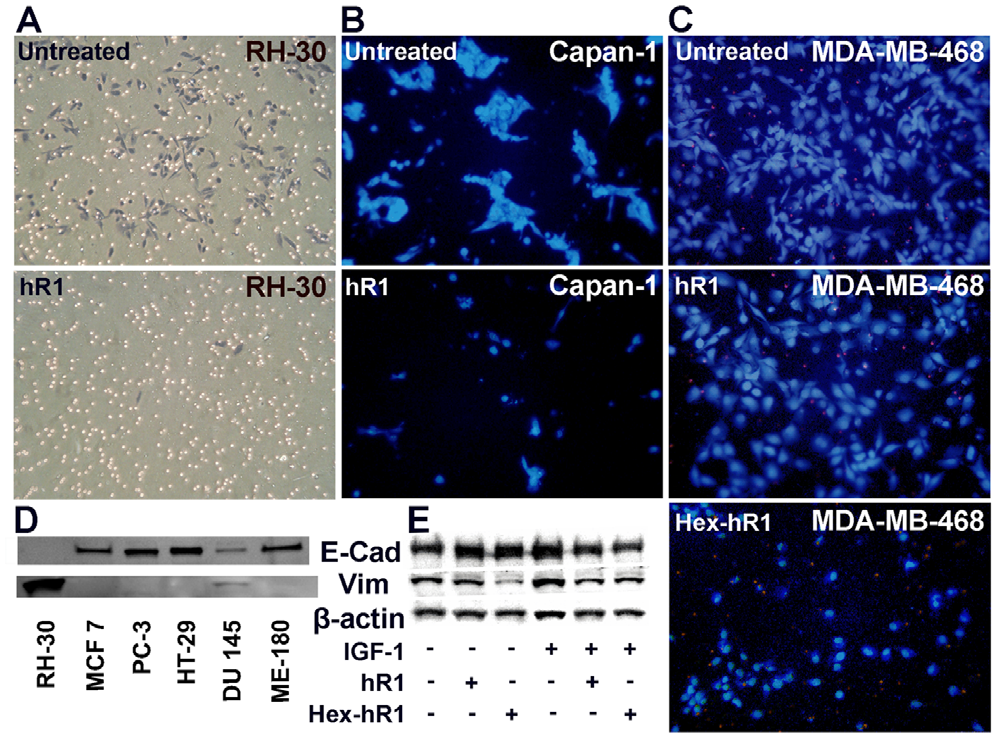
Inhibition of in vitro invasion observed for (A) RH-30 cells treated with 10 µg/mL of hR1; **(B) Capan-1 cells treated with 100 µg/mL of hR1; (C) MDA-MB-468 cells treated 100 µg/mL of hR1or Hex-hR1; (D) expression levels of E-cad and vim in RH-30, MCF7, PC-3, HT-29, DU 145 and ME-180 cells; (E) Western blot analysis of E-cad and vim in DU 145 cells following treatment with hR1 and Hex-hR1 at 100 nM in the absence or presence of IGF-1.** Cells were stained with Wright-Giemsa stain (A) or Hoechst 33258 (B and C). β-Actin was used as a loading control in (E).

In vitro invasion of RH-30 was significantly reduced by hR1 at 10 µg/mL (Fig. 6A), as was Capan-1 by hR1 at 100 µg/mL (Fig. 6B). In MDA-MB-468, Hex-hR1, but not hR1, appeared to have some inhibitory activity when tested at 100 µg/mL (Fig. 6C). To investigate whether tumor cells treated with hR1 or Hex-hR1 would invoke mesenchymal-epithelial transition (MET), the basal levels of E-cadherin (E-cad) and vimentin (vim) were determined by Western blot in a variety of solid cancer cell lines (Fig. 6D and data not shown), with the results indicating MCF7, MDA-MB-468, HepG2, HT-29, ME-180, and BxPC-3 express only E-cad; MDA-MB-231, A375, SK-MES-1, ACHN, 786-O, and RH-30 express only vim; and DU 145, Huh7, A549, and Capan-1 express both E-cad and vim. Of the 5 cell lines subsequently selected for the assay, there was no detectable difference of E-cad and vim between the treated and untreated cells from the studies performed in RH-30, ACHN, 786-O, and Capan-1. However, positive evidence for the occurrence of MET was obtained in DU 145 (Fig. 6E), which incurred an apparent increase of E-cad with a notable decrease of vim in cells incubated with 100 nM of hR1 (lane 2) or Hex-hR1 (lane 3) for 48 h in serum-free medium, compared to the untreated (lane 1). As expected, the addition of IGF-1 (100 ng/mL) to the serum-free medium promoted epithelial-mesenchymal transition (EMT) in untreated cells (lane 4), which were markedly reduced in cells treated with hR1 (lane 5) or Hex-hR1 (lane 6).

### Anti-tumor Activity in vivo

**Figure 7:**
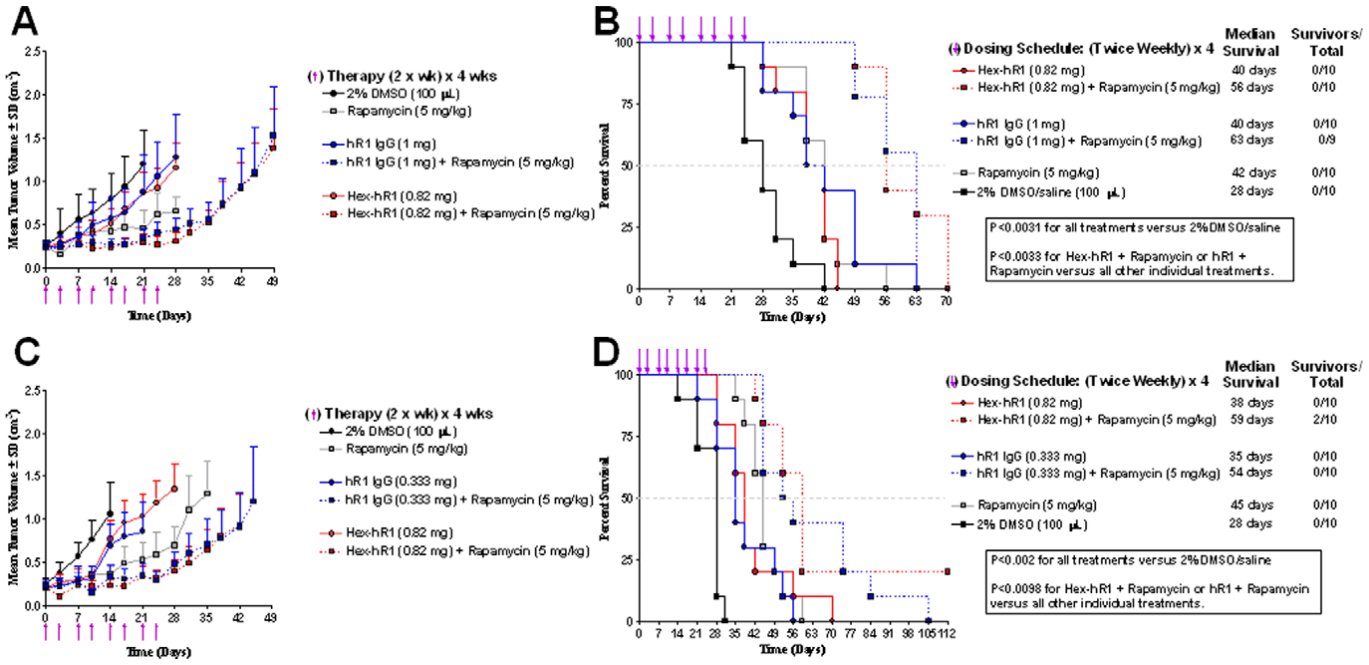
Anti-tumor activity in vivo. Mice bearing sc RH-30 tumors were administered with 100 µL 2% DMSO as control, 5 mg/kg Rapamycin, hR1-IgG and Hex-hR1 with or without Rapamycin twice weekly for 4 weeks. Tumor growth (A, C) and survival (B, D) were shown for the equimolar Fab (A and B) and protein doses (C and D).

Two different dosing regimens were examined to determine the efficacy in animals. In the first experiment, mice bearing sc RH-30 tumors were administered hR1 and Hex-hR1 at equimolar concentrations of Fab. As shown in Fig. 7A, both hR1 and Hex-hR1, when used alone, could significantly retard tumor growth in comparison to DMSO/saline control mice. After 21 days, the average tumor volumes of mice treated with hR1, Hex-hR1, and DMSA/saline, were 0.889±0.427 cm^3^, 0.876±0.230 cm^3^, and 1.208±0.393 cm^3^, respectively, with *P*<0.038 (AUC_day 21_) for the treated vs. control. When measured on day 28, the average tumor volumes of mice treated with hR1, rapamycin, and the combination of hR1 with rapamycin were 1.283±0.503 cm^3^, 0.660±0.163 cm^3^, and 0.448±0.131 cm^3^, respectively, with *P*<0.019 (AUC_day 28_) for the combination vs. single agent. These results (Fig. 7B) translated into a greater than 2-fold increase in median survival time (MST) when compared with DMSO/saline control mice (*P*<0.0001), and a greater than 50% increase in MST when compared to each agent alone (*P*<0.0033). Likewise, in mice treated with the combination of Hex-hR1 and rapamycin, the average tumor volume, when measured on day 28, was significantly smaller (0.313±0.096 cm^3^) than in mice treated with only Hex-hR1 (1.162±0.276 cm^3^) or rapamycin (0.660±0.163 cm^3^), with *P*<0.029 (AUC_day 28_) for the combination vs. single agent. This too resulted in a 2-fold increase in MST relative to DMSO/saline control mice (*P*<0.0001) and greater than 33% in comparison to each agent alone (*P*<0.0007). There were no significant differences between mice treated with hR1 plus rapamycin and those treated with Hex-hR1 plus rapamycin, both in terms of tumor growth control and survival.

In the second experiment, hR1 and Hex-hR1 were administered at equimolar concentrations of protein to address the potential impact of hexavalent binding on antitumor efficacy in vivo. As shown in Fig. 7C, both agents when used alone were effective in suppressing tumor growth relative to DMSO/saline control group. After 14 days, the average tumor volumes of mice treated with hR1, Hex-hR1, and DMSA/saline, were 0.692±0.212 cm^3^, 0.775±0.219 cm^3^, and 1.060±0.371 cm^3^, respectively, with *P*<0.0003 (AUC_day14_) for the treated vs. control. Also, the combination of hR1 and rapamycin was superior to hR1 or rapamycin alone, when comparing the average tumor volumes of respective groups on day 21 (0.341±0.150 cm^3^ vs. 0.857±0.348 cm^3^; *P = *0.003) or day 28 (0.707±0.295 cm^3^ *vs*. 1.296±0.382 cm^3^; *P = *0.0033). In terms of survival benefit, hR1 alone significantly improved median survival when compared to DMSO/saline control animals (MST = 35 days *vs*. 28 days; *P = *0.002). The addition of rapamycin to hR1 gave a further survival benefit over hR1 alone (MST = 35 days; P = 0.0035) or rapamycin alone (MST = 45 days; *P* = 0.0098). For mice treated with Hex-hR1 or Hex-hR1 plus rapamycin, the survival curves were similar to those obtained from the first experiment, indicating the reproducibility of the animal model; however, in neither experiment was an advantage of Hex-hR1 over hR1 established.

## Discussion

Antibodies against IGF-1R can be grouped into three main types, depending on the outcomes following their ligation to the receptor. Agonistic antibodies function by enhancing, or not affecting, the binding of IGFs, and in the absence of IGFs under serum-free conditions, stimulate cell proliferation and induce receptor autophosphorylation and downstream signaling, as shown for 1H7-scFv-Fc [55] and 3B7 [56]. Antagonistic antibodies, of which αIR-3 [57] and MAB391 [52] are two prominent prototypes, comprise the majority of the anti-IGF-1R antibodies and are characterized by their ability to effectively block the binding of IGFs and as a result, reduce or abrogate the stimulatory effects of IGFs, in addition to their inability to evoke receptor activation and cell proliferation. A third type, regarded as neither agonists nor antagonists and represented by 2C8 [58], would merely target IGF-1R while exerting little effect on inhibiting IGFs binding and stimulating cell proliferation. Despite these notable differences, which may be largely attributed to epitope specificity, both agonistic and antagonistic types are known to cause the degradation of IGF-1R, which has been advocated as the primary mechanism for the observed activities of various anti-IGF-1R antibodies in inhibiting anchorage-independent growth, motility, and invasion of cancer cells in vitro [39], [59], [60], as well as in reducing the colonization of xenograft tumors in metastatic animal models [61].

The results presented in this report indicate that hR1 behaved like an agonisitic anti-IGF-1R antibody, since it not only failed to inhibit IGF-1 from binding to bead-immobilized rhIGF1R, but also induced phosphorylation of IGF-1R and Akt in MCF7 cells. However, hR1 (up to 200 nM) was ineffective in stimulating the proliferation of MCF cells in serum-free medium and could moderately inhibit the stimulatory effect of IGF1 on the proliferation of RH-30 and DU 145 cells. More importantly, the colony formation of DU 145 in monolayer culture, or ACHN and 786-O cells in soft-agar, as well as the in vitro invasion of Capan-1 and RH-30 through Matrigel, was evidently reduced by hR1 when 10% serum was included in the culture medium or added as a stimulant. Moreover, downregulation of IGF-1R in MCF7, HT-29, DU 145, and LNCaP could be readily demonstrated with hR1 at 0.1 nM by Western blot. In addition, we observed in DU 145 cells that hR1 treatment resulted in a detectable change in E-cadherin and vimentin, suggesting the possible induction of MET.

The purity and molecular integrity of Hex-hR1, as well as its two modular components, C_H_1-DDD2-Fab-hR1 and C_H_3-AD2-IgG-hR1, were verified by the analytical data, particularly those of LC-MS. Both hR1 and Hex-hR1 lack antibody-dependent cellular toxicity (data not shown), and in all functional tests performed, Hex-hR1 was as active as hR1. In selective experiments where direct comparison of potency was intended, Hex-hR1 demonstrated a stronger effect on inhibiting cell proliferation stimulated by IGF-1 and downregulating IGF-1R, which was detectable for Hex-hR1 at 20 pM, as compared to 100 pM for hR1. We also noted that cells treated with Hex-hR1 consistently formed smaller colonies in soft agar than those treated with hR1.

Although Hex-hR1 demonstrated a higher potency than hR1 in inhibiting IGF-1-stimulated proliferation of RH-30 in-vitro, the current in-vivo studies indicated that Hex-hR1 and hR1 exhibited nearly equivalent efficacy in delaying growth of RH-30 xenografts when administered weekly in two different dosing regimens, one based on equimolar concentrations of Fab and the other on equimolar concentrations of protein. Inclusion of rapamycin in each dosing regimen substantially improved the survival outcomes, but the advantage of Hex-hR1 over hR1 was again not observed. Several plausible explanations for the lack of additional benefit displayed by Hex-hR1 as compared to hR1 are offered. First, the dose regimens of Hex-hR1 were not optimized in the two in-vivo studies; thus, the saturation dose of Hex-hR1, which is likely to be lower than 0.82 mg, needs to be determined and evaluated for efficacy. It is noted that the low dose of hR1 (0.33 mg) could already reach the saturation dose because it was as effective as the high dose (1 mg). Second, increasing concentrations of Hex-hR1 may prevent multiple engagements to target cells due to negative cooperativity, reducing its potency to the levels of bivalent or even monovalent binding. Third, the pharmacokinetics (Pk) is expected to be less favorable for Hex-hR1 as inferred from a previous study comparing the Pk of two bispecific HexAbs with their parental antibodies following intravenous injection in mice [62]. The two bispecific HexAbs, designated 22–20 and 20–22, are similar to Hex-hR1 in design as they also consist of a humanized IgG linked at the carboxyl terminus of each heavy chain with a stabilized dimer of Fab. Specifically, 22–20 comprises epratuzumab (anti-CD22) and four Fabs of veltuzumab (anti-CD20), whereas 20–22 comprises veltuzumab and four Fabs of epratuzumab. The elimination half-life (T_1/2_) and mean residence time (MRT) of 22–20 were determined to be 24.2 and 36.9 h, respectively, which were approximately half of the corresponding parameters found for parental antibody epratuzumab (T_1/2_ = 52.3 h, MRT = 77.8 h). For 20–22, the T_1/2_ (37.2 h) and MRT (53.6 h) were about 75% of the parental antibody veltuzumab (T_1/2_ = 45.7 h, MRT = 74.7 h). Because of the structural similarity between Hex-hR1 and either 22–20 or 20–22, as well as the fact that none of their parental antibodies (hR1, epratuzuamb, or veltuzumab) cross-react with mouse tissues, we expect Hex-hR1 to exhibit Pk in mice comparable to 22–20 or 20–22, and similarly, the Pk of hR1 to be comparable to ether epratuzumab or veltuzumab.

Of the three translational approaches to IGF-1R-targeted cancer therapy, anti-receptor antibodies and receptor tyrosine kinase inhibitors are in advanced development at various stages, and anti-ligand antibodies are rapidly gaining in interest [35], [36]. Meanwhile, the potential advantage of dual targeting to improve the efficacy and reduce side-effects is being appreciated increasingly, and pursued in multiple strategies involving bispecific antibodies or combination of antibodies aimed to simultaneously targeting two distinct epitopes of IGF-1R [63], [64], co-blocking the activation of two different receptor kinases that crosstalk with each other [16]–[19], [65], or neutralizing both IGF-1 and IGF-2 [37]. In this regard, while we are continuing to explore the therapeutic potential of Hex-hR1 and hR1, the versatility of the DNL platform also allows us to use the hR1-IgG-AD2 module to construct bispecific HexAbs [66] capable of targeting a second antigen, which may further enhance the specificity and potency of Hex-hR1 over hR1. Two such bispecific HexAbs, 1R-(E1)-(E1), comprising hR1 IgG with four Fabs of hRS7 (anti-Trop-2), and 1R-(15)-(15), comprising hR1 IgG with four Fabs of hMN-15 (anti-CEACAM6) Fab, have shown anti-invasive activity against MDA-MB-468 and Capan-1, respectively [67]. These promising results warrant further evaluation of hR1-based HexAbs for targeted therapy of solid cancers.

Due to the lack of a well-defined preclinical system that would be universally suitable for assessing anti-IGF-1R antibodies, the importance of receptor down-regulation relative to receptor inhibition/ligand blocking in affecting treatment response is yet to be determined. Encouragingly, recent endeavors have led to the identification of potential biomarkers that may be associated with the sensitivity of cancer cells to anti-IGF-1R antibodies, although a unified theme is still wanting. For example, in a survey of 41 breast and 27 colorectal cancer cell lines, the expression levels of IGF-1R, IRS-1, IRS-2, and IGF-2 were correlated with positive predictive values for h10H5 [53]. In another study involving 22 cell lines of NSCLC, high levels of total IGF-1R, but not phosphorylated IGF-1R, were identified as one potential biomarker of sensitivity to R1507 [54]. Analysis of 9 rhabdomyosarcoma cell lines also revealed a direct and very significant correlation between elevated IGF-IR levels and antiproliferative effects of h7C10 and defined a minimal receptor number (>3000) that would predict sensitivity [51]. Thus, we anticipate that hR1 and Hex-hR1 would display further comparability with their antagonistic rivals in a more refined setting where predictive biomarkers can be clearly validated. Nevertheless, it is noteworthy that the 30 to 50% growth inhibition of DU 145 and ME-180 cells in IGF-1-containing medium by Hex-hR1 was already comparable to the 25 to 50% growth inhibition of “drug-sensitive” NSCLC lines achieved with R1507, a representative ligand-blocking, antagonistic anti-IGF-1R human antibody [54]. In such assays, Hex-hR1 was more potent than hR1 in RH-30 and DU 145 cells, with EC_50_ values more than 25-fold lower.

## Supporting Information

Figure S1 Figure S1A: The amino acid sequence of hR1 VH. Figure S1B: The amino acid sequence of hR1 Vκ.
(PPT)

Figure S2 **Competition binding of mR1 or cR1 vs. Alexa-R1.**
(PPT)

Figure S3 **Binding Affinity of cR1 to immobilized rhIGF-1R.**
(PPT)

Figure S4 **Binding of cR1 to immobilized rhIGF-1R, but not to rhIR.**
(PPT)

Figure S5 **Competition binding of cR1 vs. IGF-1 or IGF-2.**
(PPT)

Figure S6 **Competition binding of R1, cR1, or hR1 vs. Alexa-cR1.**
(PPT)

Figure S7 **Competition binding of R1, hR1, MAB391 or IGF-1 vs. radioiodinated IGF-1.**
(PPT)

Figure S8 Figure S8A: Competition binding of R1 or MAB391 vs. PE-R1. Figure S8B: Competition binding of R1 or MAB391 vs. PE-MAB391.
(PPT)

Figure S9 **Downregulation of cell surface IGF-1R as determined by flow cytometry in MCF7 and DU 145 following overnight treatment with hR1 or Hex-hR1 at 10 nM.**
(PPT)

Table S1 **Key properties of published anti-IGF-1R antibodies (References attached).**
(DOC)

Table S2 **N-terminal protein sequencing of R1.**
(DOC)

Table S3 **Binding of ^125^I-IGF-1 to MCF-7L in the presence of MAB391 or R1.**
(DOC)
