# Supplementary material for: Evaluation of a Novel Hexavalent Humanized Anti-IGF-1R Antibody and Its Bivalent Parental IgG in Diverse Cancer Cell Lines
Source: PLoS One. 2012 Aug 31;7(8):e44235. doi: 10.1371/journal.pone.0044235 (PMC3432068; doi:10.1371/journal.pone.0044235)
Supplement: Figure S1 — A: The amino acid sequence of hR1 VH. Figure S1B: The amino acid sequence of hR1 Vκ. (PPT) [file pone.0044235.s001.ppt]

## Slide 1
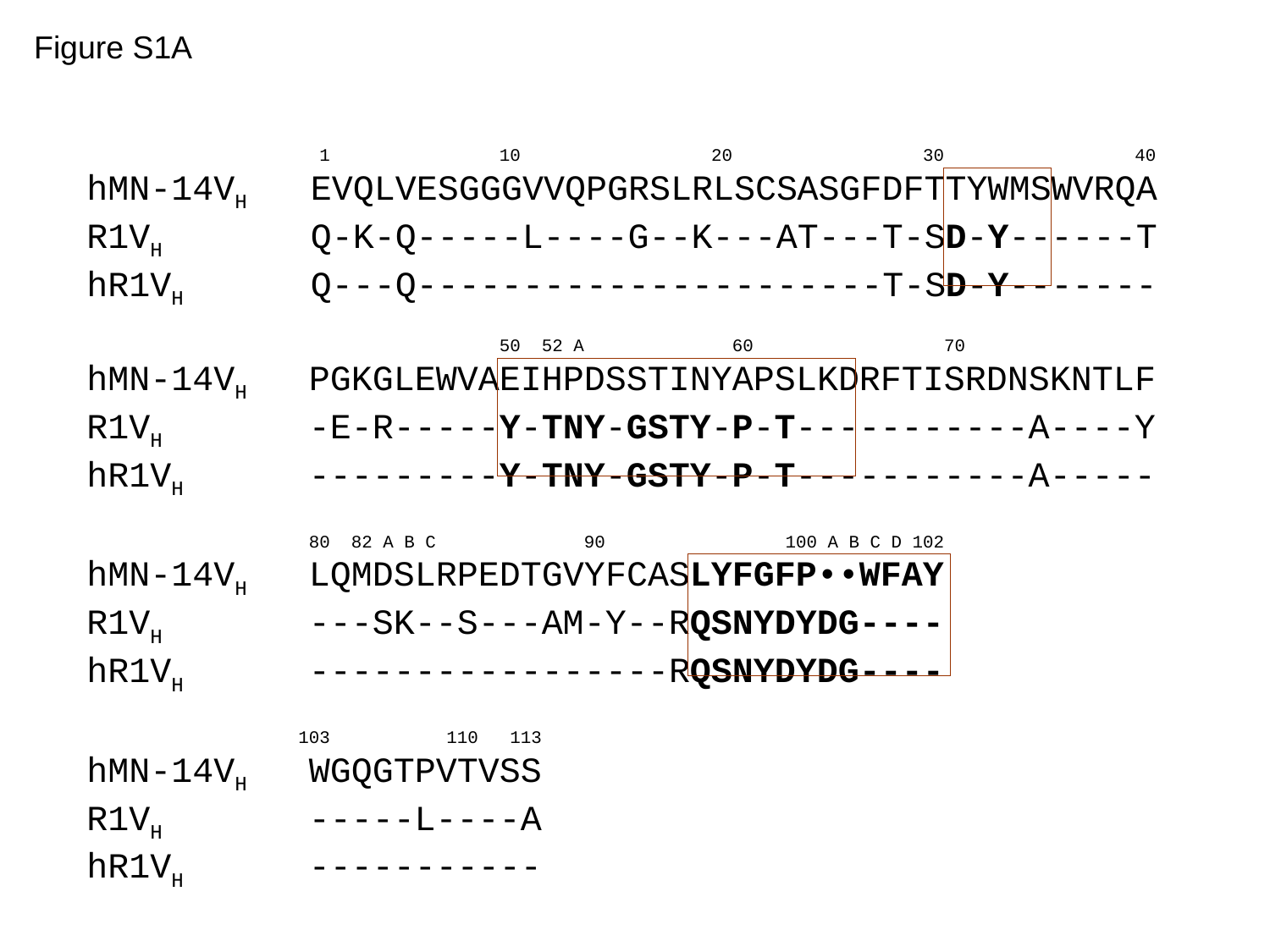

Figure S1A
 1 10 20 30 40
hMN-14VH EVQLVESGGGVVQPGRSLRLSCSASGFDFTTYWMSWVRQA
R1VH Q-K-Q-----L----G--K---AT---T-SD-Y------T
hR1VH Q---Q----------------------T-SD-Y-------
 50 52 A 60 70
hMN-14VH 	PGKGLEWVAEIHPDSSTINYAPSLKDRFTISRDNSKNTLF
R1VH 	-E-R-----Y-TNY-GSTY-P-T-----------A----Y hR1VH 	---------Y-TNY-GSTY-P-T-----------A-----
 80 82 A B C 90 100 A B C D 102
hMN-14VH 	LQMDSLRPEDTGVYFCASLYFGFP••WFAY
R1VH 	---SK--S---AM-Y--RQSNYDYDG----
hR1VH 	-----------------RQSNYDYDG----
 103 110 113
hMN-14VH 	WGQGTPVTVSS
R1VH 	-----L----A
hR1VH 	-----------

## Slide 2
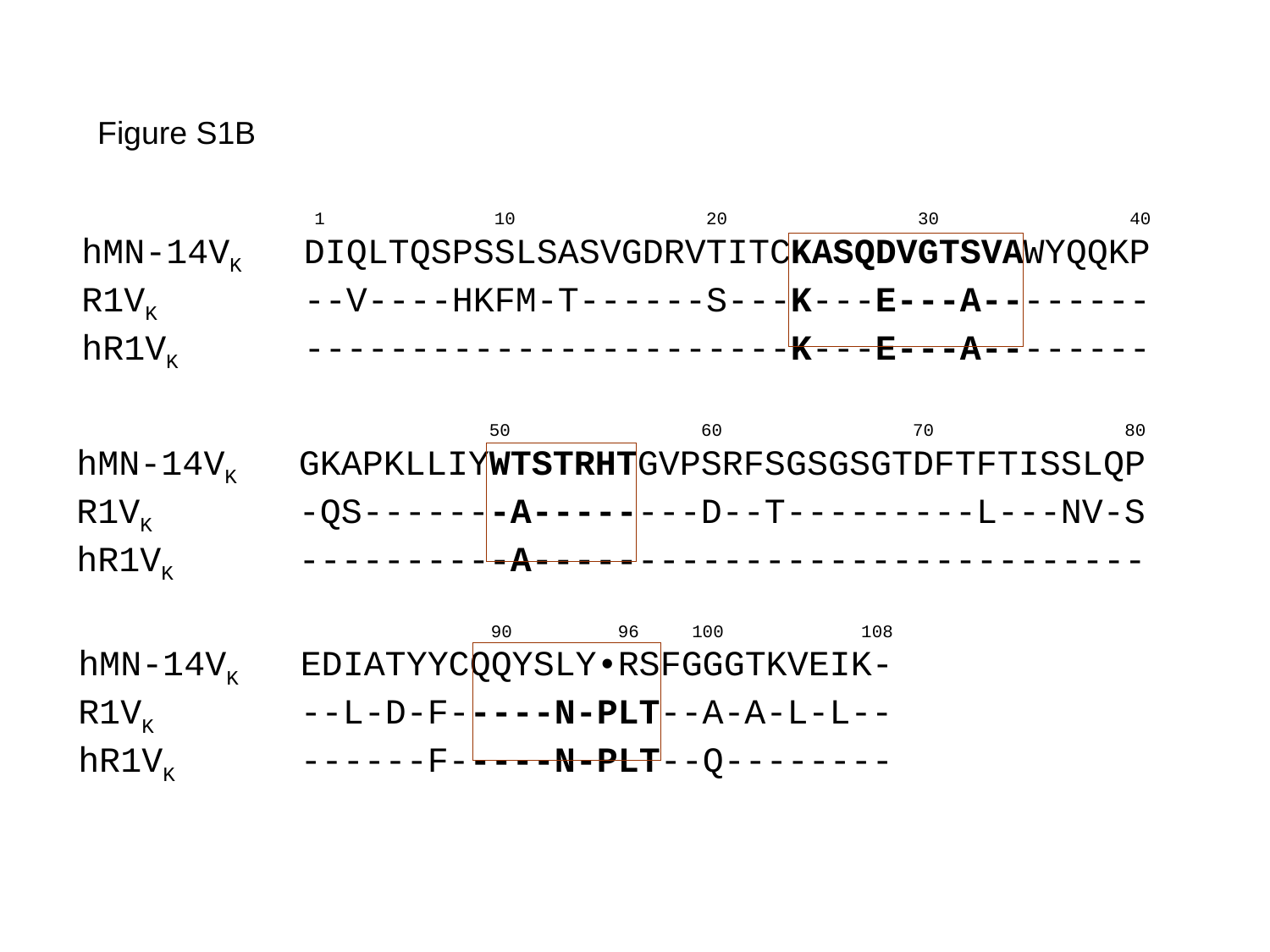

Figure S1B
 1 10 20 30 40
hMN-14VK 	DIQLTQSPSSLSASVGDRVTITCKASQDVGTSVAWYQQKP
R1VK 	--V----HKFM-T------S---K---E---A-------- hR1VK 	-----------------------K---E---A--------
 50 60 70 80
hMN-14VK 	GKAPKLLIYWTSTRHTGVPSRFSGSGSGTDFTFTISSLQP
R1VK 	-QS-------A--------D--T---------L---NV-S
hR1VK 	----------A-----------------------------
 90 96 100 108
hMN-14VK 	EDIATYYCQQYSLY•RSFGGGTKVEIK-
R1VK 	--L-D-F-----N-PLT--A-A-L-L--
hR1VK 	------F-----N-PLT--Q--------
