# Supplementary figures and images for: Evaluation of a Novel Hexavalent Humanized Anti-IGF-1R Antibody and Its Bivalent Parental IgG in Diverse Cancer Cell Lines
Source: PLoS One. 2012 Aug 31;7(8):e44235. doi: 10.1371/journal.pone.0044235 (PMC3432068; doi:10.1371/journal.pone.0044235)

## Slide 1
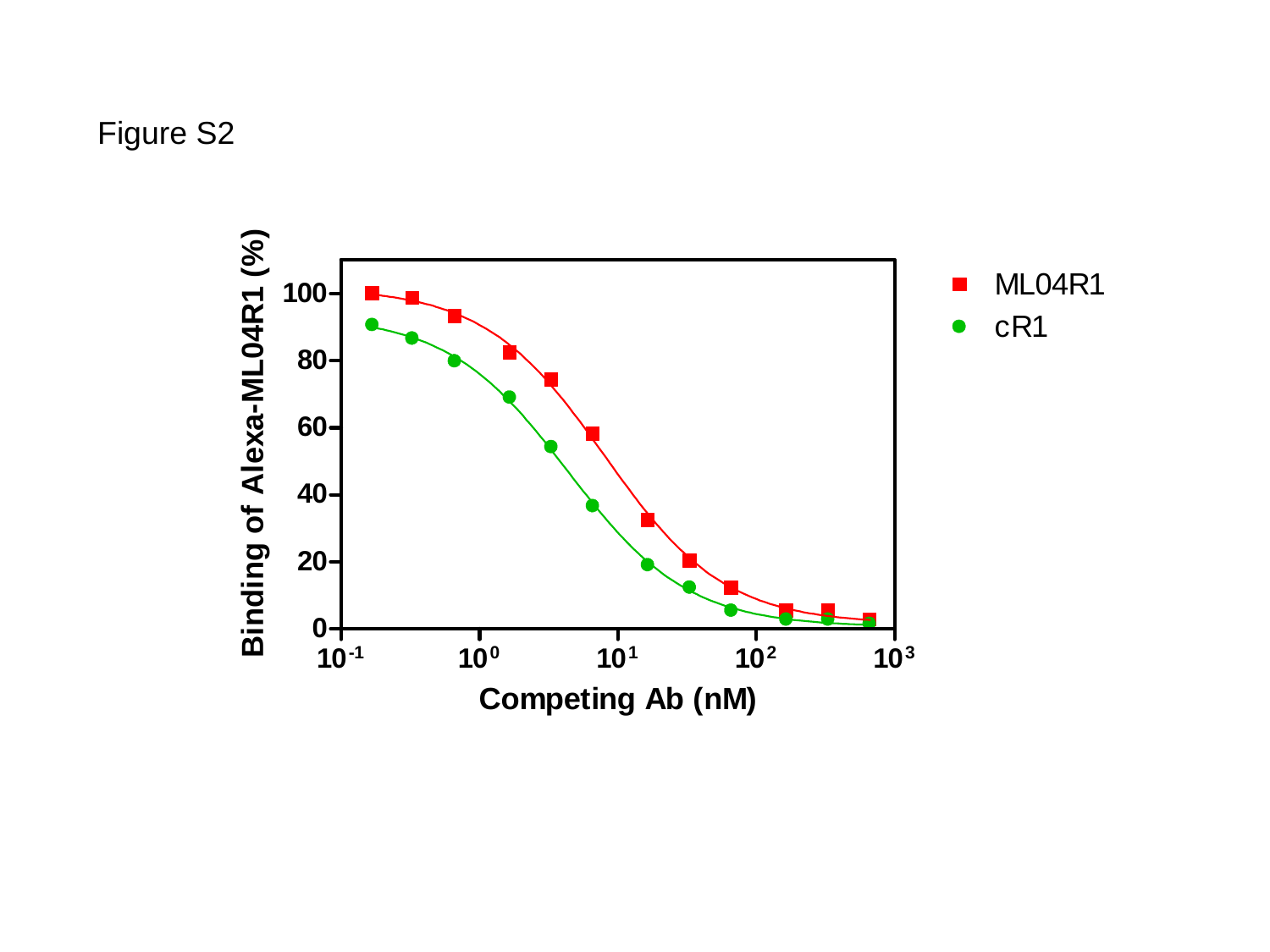

Figure S2

Supplement: Figure S2 — Competition binding of mR1 or cR1 vs. Alexa-R1. (PPT) [file pone.0044235.s002.ppt]

## Slide 1
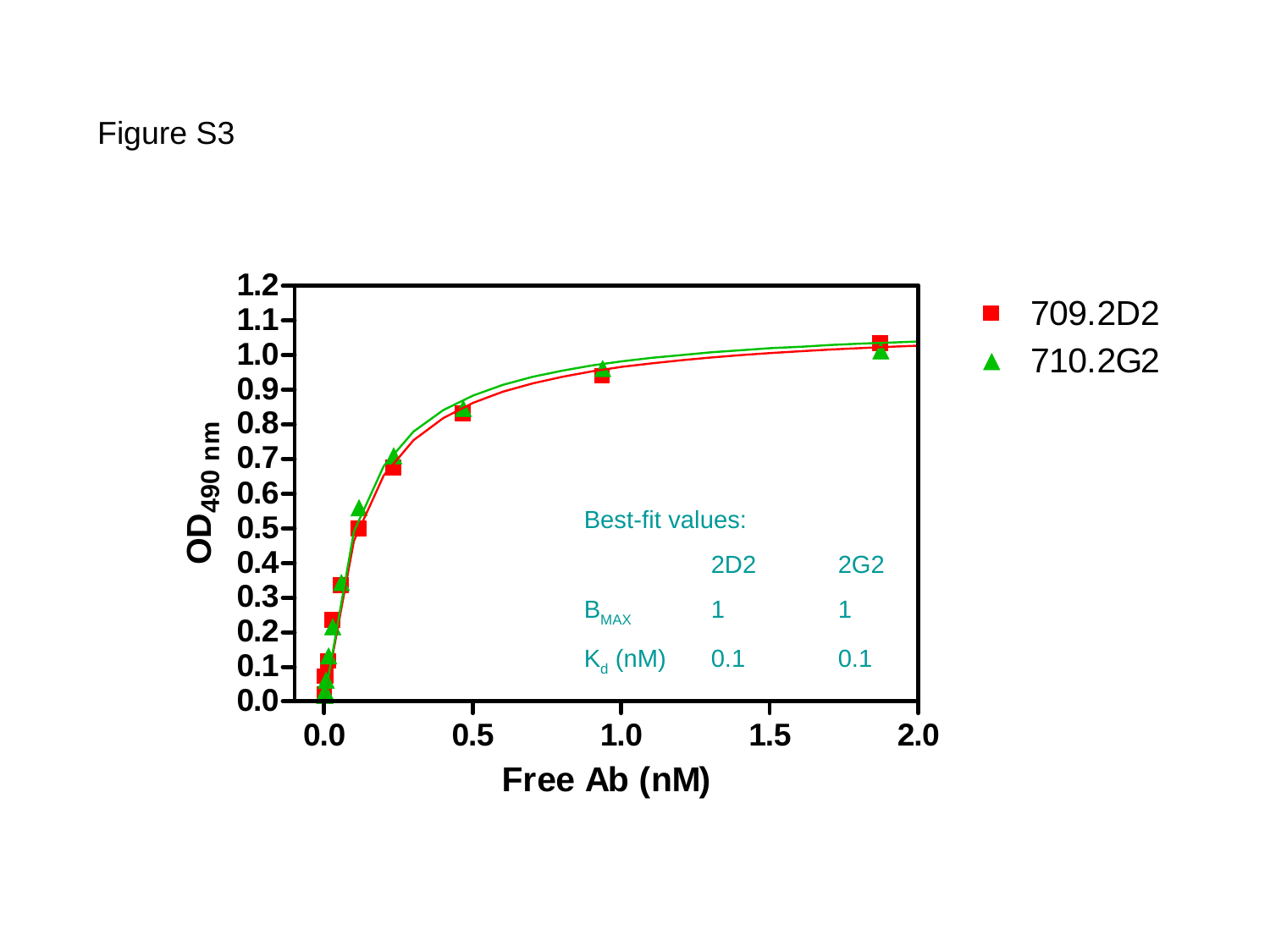

Figure S3
Best-fit values:
	2D2	2G2
BMAX	1	1
Kd (nM)	0.1	0.1
Confidential

Supplement: Figure S3 — Binding Affinity of cR1 to immobilized rhIGF-1R. (PPT) [file pone.0044235.s003.ppt]

## Slide 1
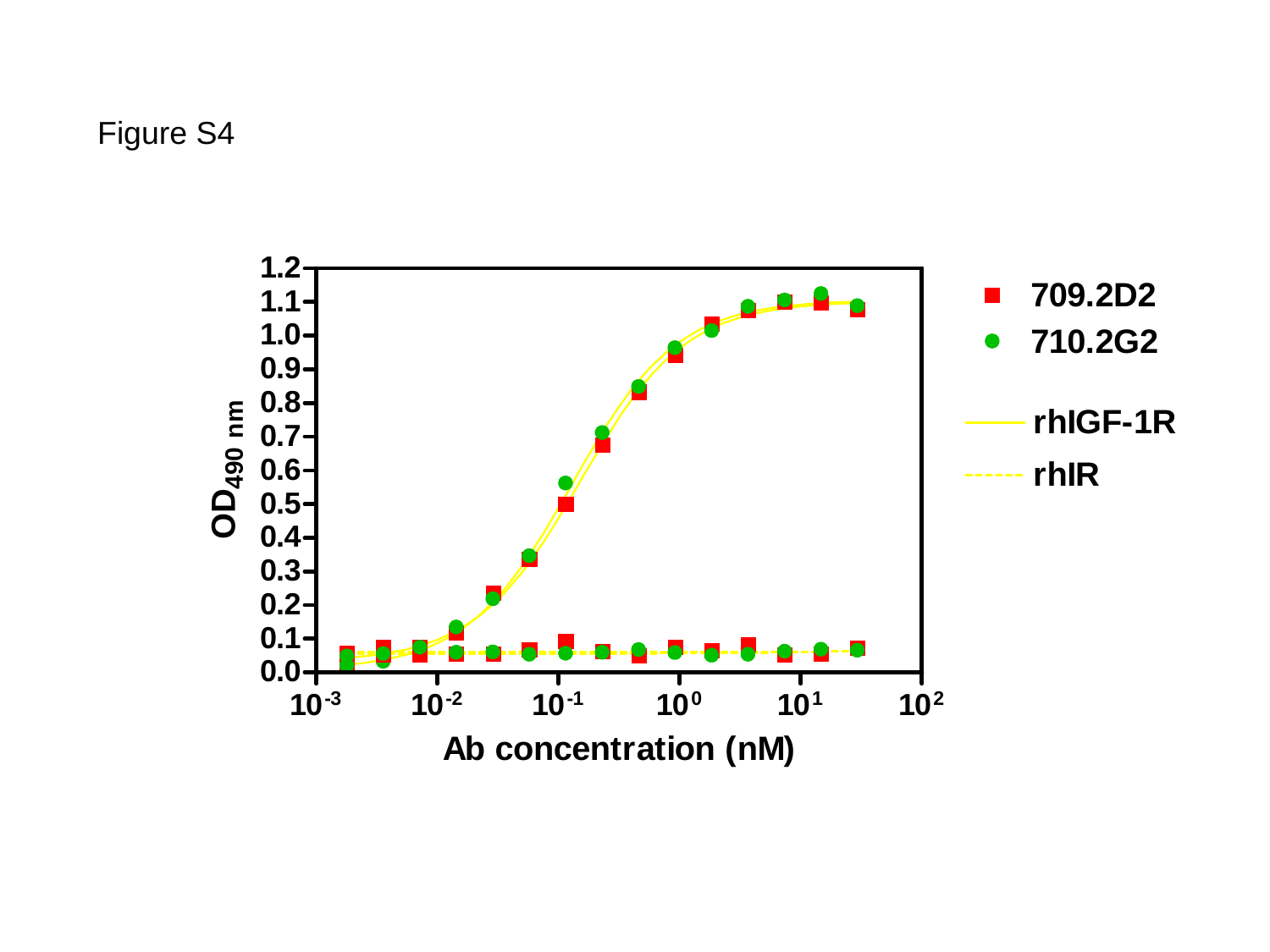

Figure S4
Confidential

Supplement: Figure S4 — Binding of cR1 to immobilized rhIGF-1R, but not to rhIR. (PPT) [file pone.0044235.s004.ppt]

## Slide 1
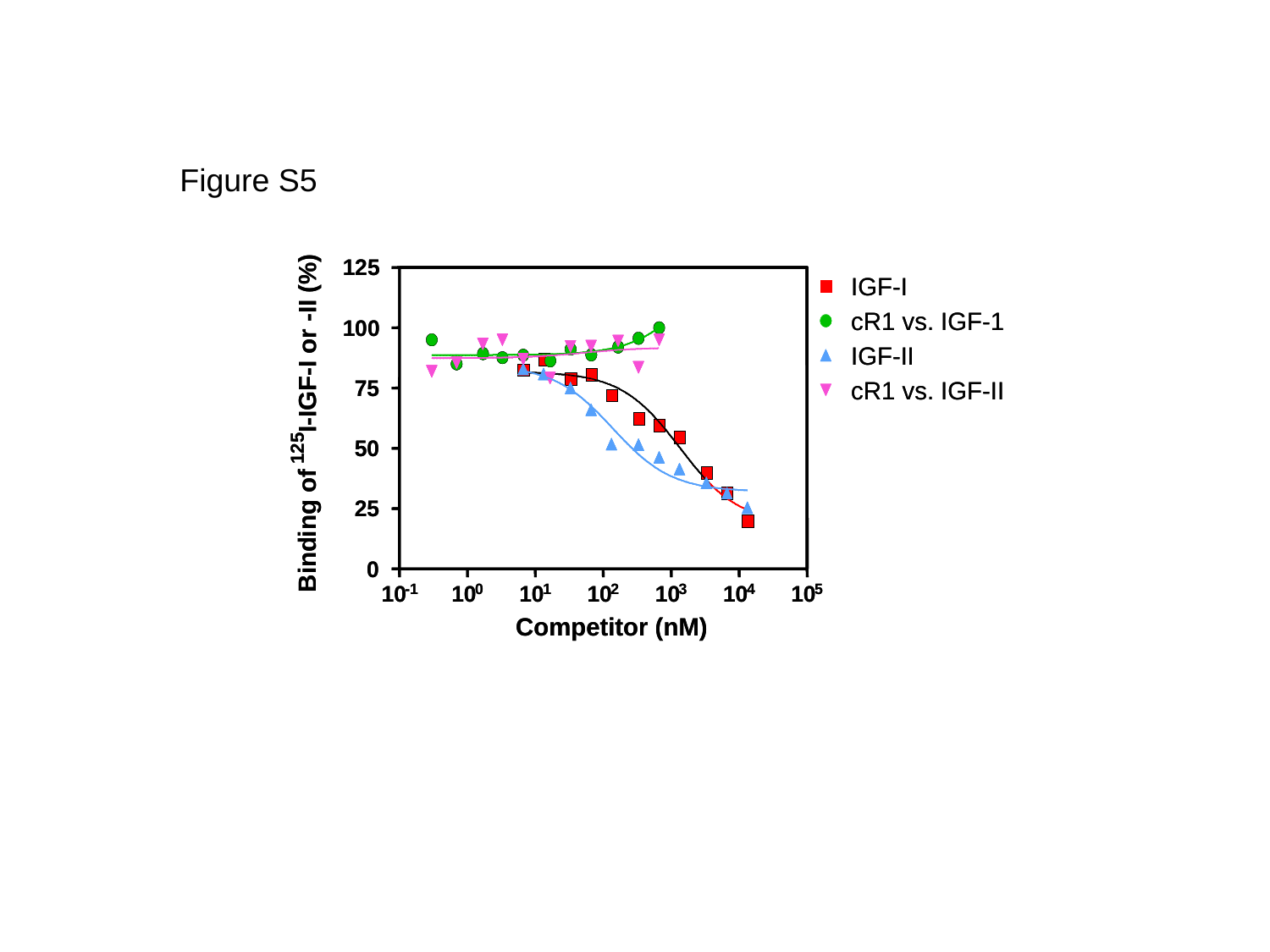

Figure S5

Supplement: Figure S5 — Competition binding of cR1 vs. IGF-1 or IGF-2. (PPT) [file pone.0044235.s005.ppt]

## Slide 1
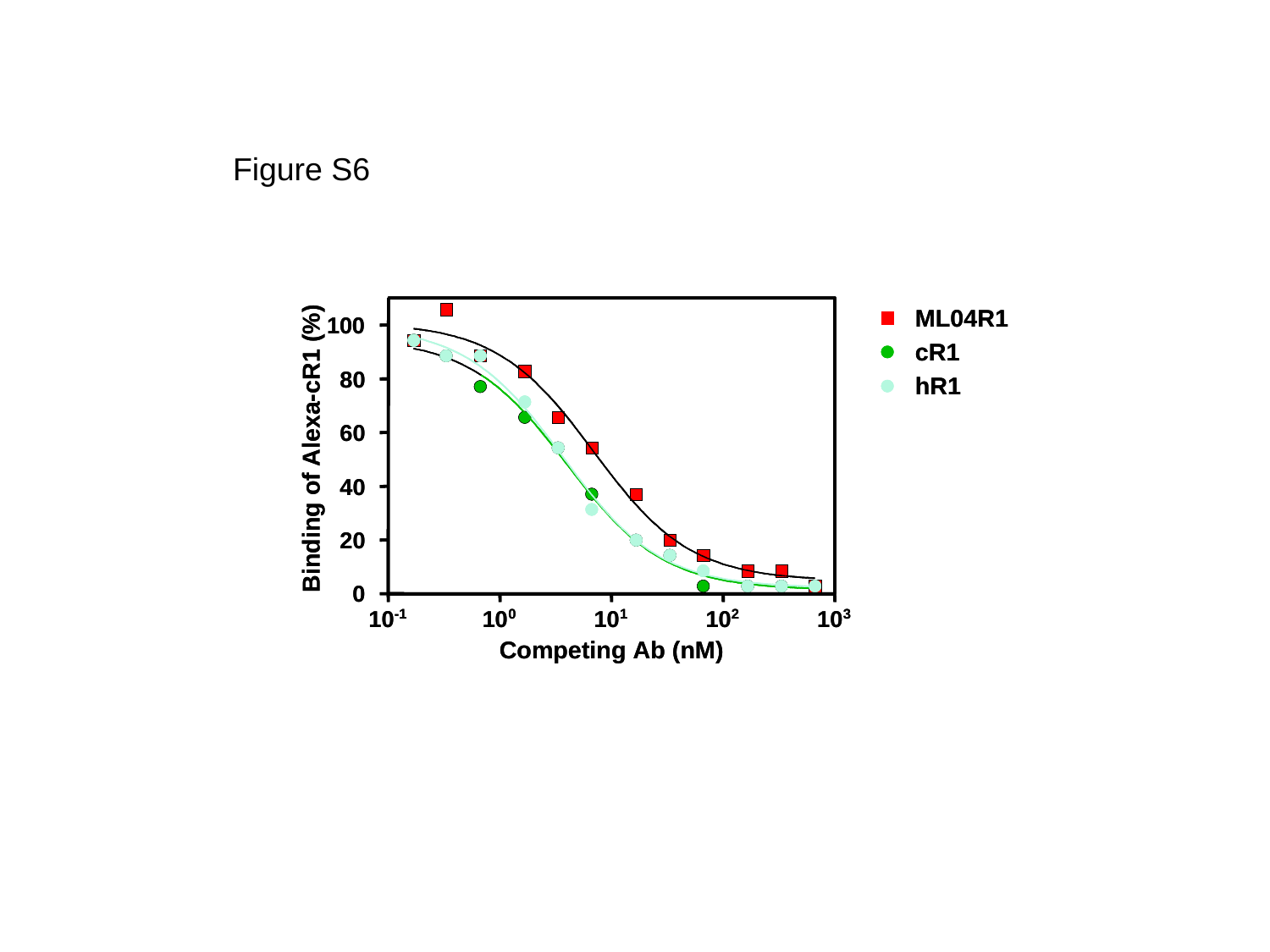

Figure S6

Supplement: Figure S6 — Competition binding of R1, cR1, or hR1 vs. Alexa-cR1. (PPT) [file pone.0044235.s006.ppt]

## Slide 1
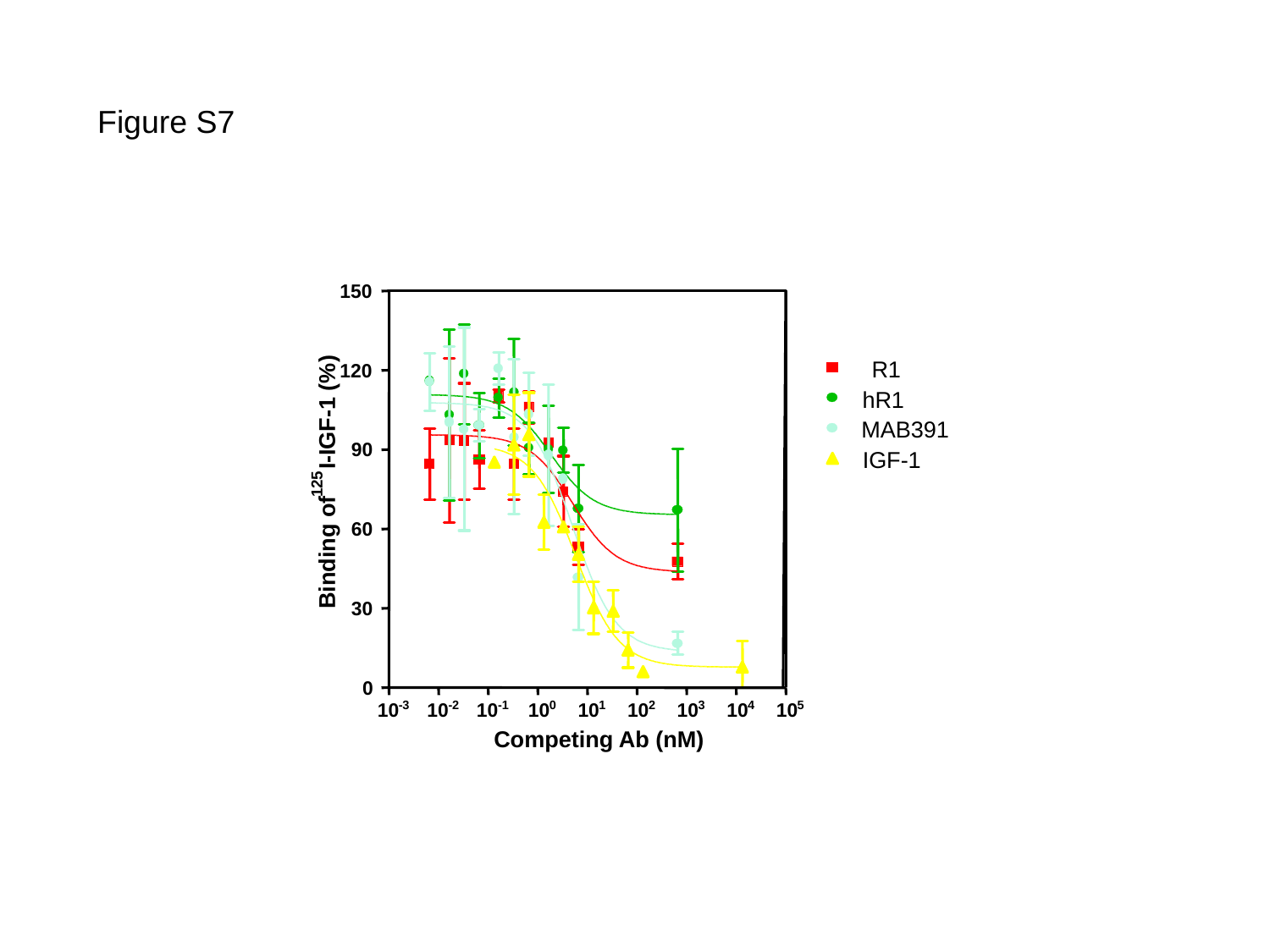

Figure S7
150
R1
120
hR1
I-IGF-1 (%)
MAB391
90
IGF-1
125
60
Binding of
30
0
10
-3
10
-2
10
-1
10
0
10
1
10
2
10
3
10
4
10
5
Competing Ab (nM)

Supplement: Figure S7 — Competition binding of R1, hR1, MAB391 or IGF-1 vs. radioiodinated IGF-1. (PPT) [file pone.0044235.s007.ppt]
