# Supplementary material for: Evaluation of a Novel Hexavalent Humanized Anti-IGF-1R Antibody and Its Bivalent Parental IgG in Diverse Cancer Cell Lines
Source: PLoS One. 2012 Aug 31;7(8):e44235. doi: 10.1371/journal.pone.0044235 (PMC3432068; doi:10.1371/journal.pone.0044235)
Supplement: Figure S8 — A: Competition binding of R1 or MAB391 vs. PE-R1. Figure S8B: Competition binding of R1 or MAB391 vs. PE-MAB391. (PPT) [file pone.0044235.s008.ppt]

## Slide 1
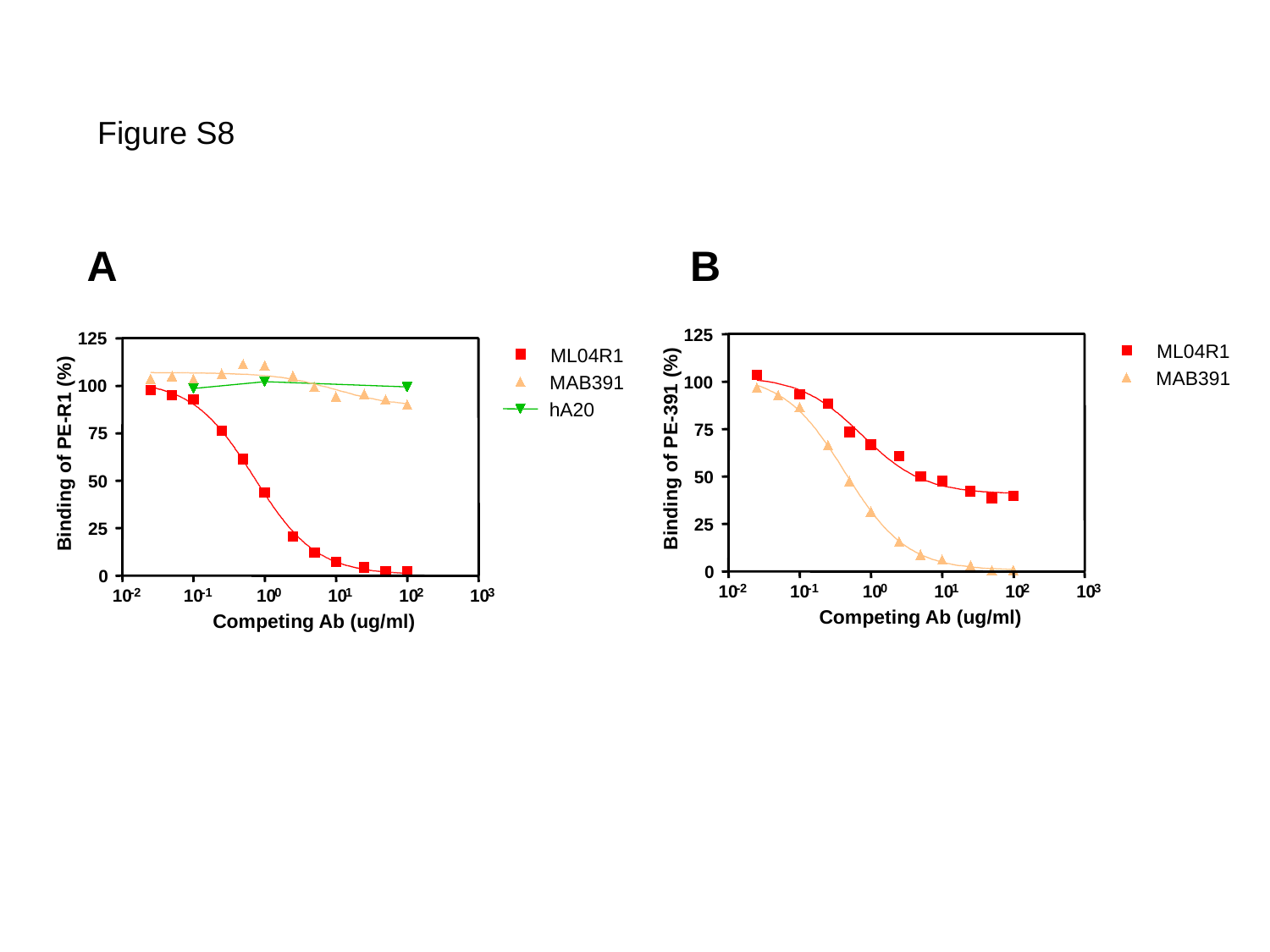

Figure S8
A
B
125
125
ML04R1
MAB391
100
hA20
75
Binding of PE-R1 (%)
50
25
0
10
-2
10
-1
10
0
10
1
10
2
10
3
Competing Ab (ug/ml)
ML04R1
MAB391
100
10
-2
10
-1
75
Binding of PE-391 (%)
50
25
0
10
0
10
1
10
2
10
3
Competing Ab (ug/ml)
