# Supplementary material for: Evaluation of a Novel Hexavalent Humanized Anti-IGF-1R Antibody and Its Bivalent Parental IgG in Diverse Cancer Cell Lines
Source: PLoS One. 2012 Aug 31;7(8):e44235. doi: 10.1371/journal.pone.0044235 (PMC3432068; doi:10.1371/journal.pone.0044235)
Supplement: Figure S9 — Downregulation of cell surface IGF-1R as determined by flow cytometry in MCF7 and DU 145 following overnight treatment with hR1 or Hex-hR1 at 10 nM. (PPT) [file pone.0044235.s009.ppt]

## Slide 1
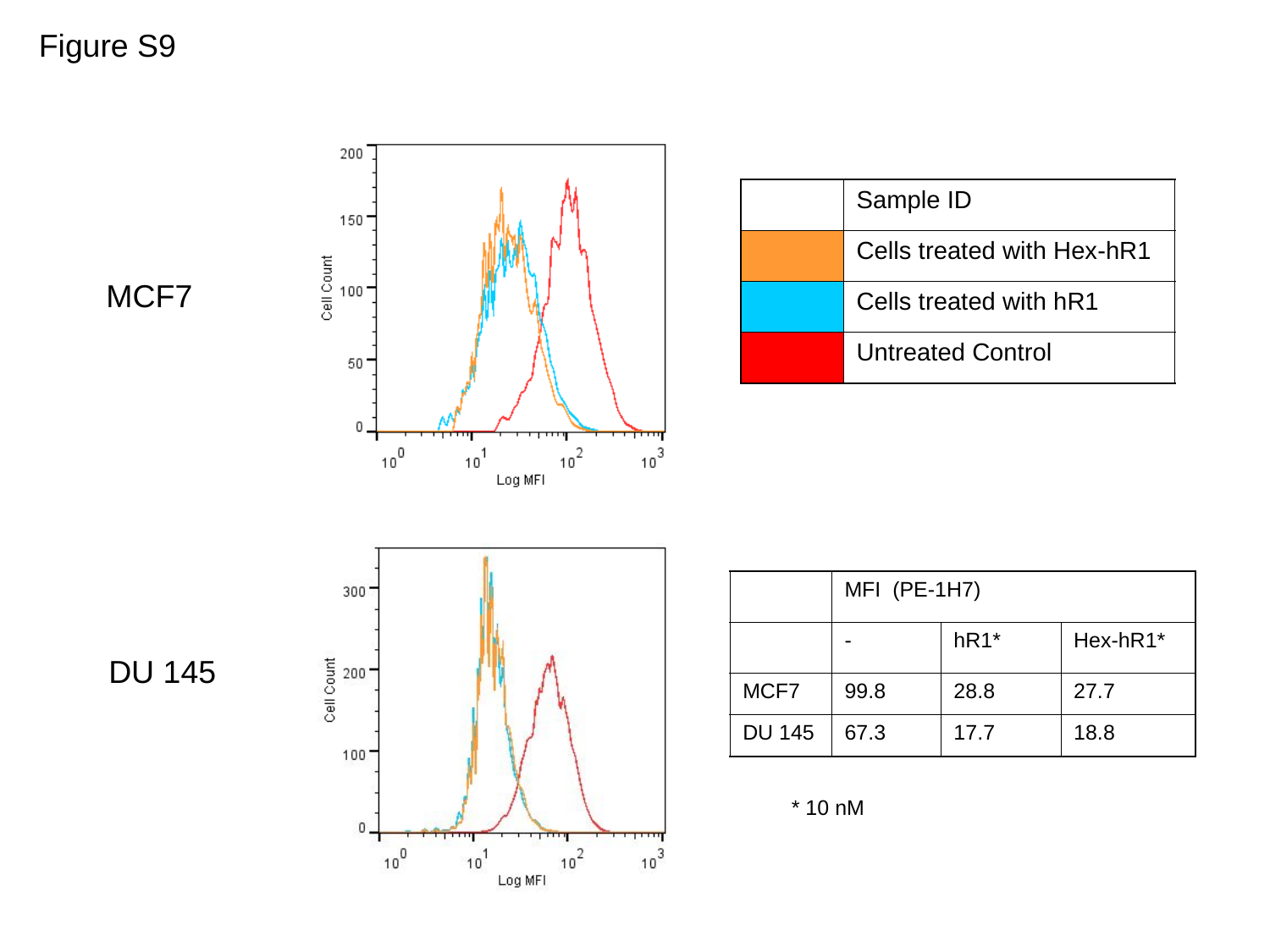

Figure S9
| | Sample ID |
| --- | --- |
| | Cells treated with Hex-hR1 |
| | Cells treated with hR1 |
| | Untreated Control |
MCF7
| | MFI (PE-1H7) | | |
| --- | --- | --- | --- |
| | - | hR1\* | Hex-hR1\* |
| MCF7 | 99.8 | 28.8 | 27.7 |
| DU 145 | 67.3 | 17.7 | 18.8 |
DU 145
* 10 nM
