# Supplementary material for: Evaluation of a Novel Hexavalent Humanized Anti-IGF-1R Antibody and Its Bivalent Parental IgG in Diverse Cancer Cell Lines
Source: PLoS One. 2012 Aug 31;7(8):e44235. doi: 10.1371/journal.pone.0044235 (PMC3432068; doi:10.1371/journal.pone.0044235)
Supplement: Table S1 — Key properties of published anti-IGF-1R antibodies (References attached). (DOC) [file pone.0044235.s010.doc]

Table S1. Key properties of published anti-IGF-1R mAbs

| mAb | Species | Isotype | K_D_ (nM)^a^ | Ligand binding^b^ | | Epitope(s)^c, d^ | Ref^e^ |
| --- | --- | --- | --- | --- | --- | --- | --- |
|  |  |  | IGF-1R | IGF-1 | IGF-2 |  |  |
| 3B7 | mouse | IgG1 | 8.6 | ↑ | ↑ | 62-184 | S1 |
| 4-52 | mouse | IgG1 | ?^f^ | ↑ | ? | 62-184 | S2 |
| 16-13 | mouse | IgG2b | ? | ↑ | ? | 62-184 | S2 |
| 24-31 | mouse | IgG1 | ? | ↔ | ? | 283-440 | S2, S3 |
| 17-69 | mouse | IgG1 | ? | ↓ | ? | 514-586 | S2, S3 |
| 26-3 | mouse | IgG1 | ? | ↑ | ? | 283-440 | S2, S3 |
| 24-55 | mouse | IgG1 | ? | ↓ | ? | 440-514 | S2, S3 |
| 24-57 | mouse | IgG1 | ? | ↓ | ? | 440-514 | S2, S3 |
| 24-60 | mouse | IgG2a | ? | ↓ | ↔ ^g^ | 184-283 | S2, S3 |
| αIR-3 | mouse | IgG1 | 1.3^g^ | ↓ | ↔ | 223-274 | S4, S5 |
| MAB391 | mouse | IgG1 | ? | ↓ | ↔ | ND | S6 |
| 1H7 | mouse | IgG1 | 1 | ↓ | ↓ | 440-514 | S1, S7 |
| 2C8 | mouse | IgG1 | ? | ↔ | ↔ | ? | S7 |
| EM164 | mouse | IgG1 | 0.1 | ↓ | ? | 1-458/691-706 | S8 |
| 7C2 | mouse | IgG1 | 0.5 | ↓ | ↔ | 131-315 | S9 |
| 9E11 | mouse | IgG1 | 2.1 | ↓ | ↔ | 131-315 | S9 |
| BIIB1 | chimeric | IgG4 | ? | ↓ | ↔ | 1-462 | S10 |
| BIIB2 | mouse | ? | ? | ↔ | ↓ | 1-462 | S10 |
| BIIB3 | chimeric | IgG4 | ? | ↓ | ↓ | 1-462 | S10 |
| BIIB4 | human | IgG4 | 4 | ↓ | ↓ | 1-462 | S10 |
| BIIB5 | human | IgG4 | 1 | ↓ | ↓ | 461-579 | S10 |
| BIIB022^h^ | human | IgG4 | 1 | ↓ | ↓ | 461-579 | S11 |
| SCH-717454 | human | IgG1 | 0.004 | ↓ | ? | ? | S12 |
| IMC-A12 | human | IgG1 | 0.04 | ↓ | ↓ | ? | S13 |
| AMG479 | human | IgG1 | 0.33 | ↓ | ↓ | ? | S14 |
| CP-751,871 | human | IgG2 | 1.5 | ↓ | ↓ | ? | S15 |
| R1507 | human | IgG1 | 5 | ↓ | ↓ | ? | S16 |
| AVE1642^i^ | humanized | IgG1 | 0.1 | ↓ | ↓ | 1-458/691-706 | S17 |
| h7C10 | humanized | IgG1 | ? | ↓ | ↓ | ? | S18 |
| h10H5 | humanized | IgG1 | 0.2 | ↓ | ↓ | ? | S19 |
| m590 | chimeric | IgG1 | ? | ↓ | ↓ | ? | S20 |

^a^Dissociation constant as reported in the cited reference.

^b^Enhancement (↑); inhibition (↓); no effect (↔).

^c^ The amino acid residues involved in the antibody binding as reported in the cited reference.

^d^The α-chain of IGF-1R comprises L1 (1-150), CR (151-299), L2 (300-460), FnIII-1 (461-579), and FnIII-2 (580-707).

^e^ Representative publication(s) only.

^f^Information not available.

^g^As reported in S9.

^h^Presumably the nonglycosylated form of BIIB5.

^i^Humanized form of EM164.

**References (Table S1)**

S1. Kusada Y, Morizono T, Matsumoto-Takasaki A, Sakai K, Sato S, et al. (2008) Construction and characterization of single-chain antibodies against human insulin-like growth factor-I receptor from hybridomas producing 1H7 or 3B7 monoclonal antibodies. J Biochem (Tokyo) 143: 9-19.

S2. [Soos MA](http://www.ncbi.nlm.nih.gov/pubmed?term="Soos MA"%5BAuthor%5D), Field CE, Lammers R, Ullrich A, Zhang B, et al (1992) A panel of monoclonal antibodies for the type I insulin-like growth factor receptor. Epitope mapping, effects on ligand binding, and biological activity. J Biol Chem 267: 12955-12963.

S3. Schumacher R, Soos MA*,* Schlessinger J, Brandenburg D, Siddle K, et al. (1993) Signaling-competent receptor chimeras allow mapping of major insulin receptor binding domain determinants. J Biol Chem 268: 1087-1094.

S4. Kull FC, Jr, Jacobs S, Su Y-F, Svoboda ME, van Wykj JJ (1983) Monoclonal antibodies to receptors for **i**nsulin and somatomedin-C. J Biol Chem258: 6561-6566.

S5. Gustafson TA, Rutter WJ (1990) The cysteine-rich domains of the insulin and insulin-like growth factor I receptors are primary determinants of hormone binding specificity. Evidence from receptor chimeras. J Biol Chem 265: 18663-18667.

S6. Hailey J, Maxwell E, Koukouras K, Bishop WR, Pachter JA, et al. (2002) Neutralizing anti-insulin-like growth factor receptor 1 antibodies Inhibit receptor function and induce receptor degradation in tumor cells. Mol Cancer Ther 1: 1349-1353.

S7. Li S-I, Kato J, Paz B, Kasuya J, Fujita-Yamaguchi Y (1992) Two new monoclonal antibodies against the α subunit of the human insulin-like growth factor-1 receptor. Biochem Biophys Res Commun 196: 92-98.

S8. Maloney EK, McLaughlin JL, Dagdigian NE, Garrett LM, Connors KM, et al. (2003) An anti-insulin-like growth factor I receptor antibody that is a potent inhibitor of cancer cell proliferation. Cancer Res 63: 5073–5083.

S9. Keyhanfar M, Booker GW, Whittaker J, Wallace JC, Forbes BE (2007) Precise mapping of an IGF-1-binding site on IGF-1R. Biochem J 401: 269-277.

S10. Doern A, Cao X, Sereno A, Reyes CL, Altshuler A, et al. (2009) Characterization of inhibitory anti-insulin-like growth factor receptor antibodies with different epitope specificity and ligand-blocking properties. J Biol Chem 284: 10254-10267.

S11. Von Mehren M, Britten C, Lear K, Camidge DR, Wainberg ZA, et al. (2010) Phase I, dose-escalation study of BIIB022 (anti-IGF-1R antibody) in advanced solid tumors. J. Clin Oncol 28 (May 20 supplement): 2612.

S12. Wang Y, Hailey J, Williams D, Wang Y, Lipari P, et al. (2005) Inhibition of insulin-like growth factor-I receptor (IGF-IR) signaling and tumor cell growth by a fully human neutralizing anti–IGF-IR antibody. Mol Cancer Ther 4: 1214-1221.

S13. Burtrum D, Zhu Z, Lu D, Anderson DM, Prewett M, et al. (2003) A fully human monoclonal antibody to the insulin-like growth factor I receptor blocks ligand-dependent signaling and inhibits human tumor growth *in vivo*. Cancer Res 63: 8912-8921.

S14 Beltran PJ, Mitchell P, Chung Y-A, Cajulis E, Lu J, et al. (2009) AMG 479, a fully human anti-insulin-like growth factorof pancreatic carcinoma cells receptor type I monoclonal antibody, inhibits the growth and survival of pancreatic carcinoma cells. Mol Cancer Ther 8: 1095-1105.

S15. Cohen BD, Baker DA, Soderstrom C, Tkalcevic T, Ann Marie Rossi AM (2005) Combination therapy enhances the inhibition of tumor growth with the fully human anti–type 1 insulin-like growth factor receptor monoclonal antibody CP-751,871. Clin Cancer Res 11:2063-2073.

S16 Croasdale R, Wartha K, Schanzer JM, Klaus-Peter Kuenkele K-P, Ries C, et al. (2012) Development of tetravalent IgG1 dual targeting IGF-1R–EGFR antibodies with potent tumor inhibition. Arch Biochem Biophys March 21 [Epub ahead of print].

S17 Desbois-Mouthon C, Baron A, Blivet-Van Eggelpoel M-J, Fartoux L, Venot C, et al. (2009) Insulin-like growth factor-1 receptor inhibition induces a resistance mechanism via the epidermal growth factor receptor/HER3/AKT signaling pathway: rational basis for cotargeting Insulin-iike growth factor-1 receptor and epidermal growth factor receptor in hepatocellular carcinoma. Clin Cancer Res 15: 5445-5456.

S18 Goetsch L, Gonzalez A, Leger O, Beck A, Pauwels PJ, et al (2005) A recombinant humanized anti-Insulin-like growth factor receptor type I antibody (h7C10) enhances the antitumor activity of vinorelbine and anti-epidermal growth factor receptor therapy against human cancer xenografts. Int. J. Cancer 113**:** 316–328.

S19 Shang Y, Mao Y, Batson J, Scales SJ, Phillips G, et al. (2008) Antixenograft tumor activity of a humanized anti-insulin-like growth factor-I receptor monoclonal antibody is associated with decreased AKT activation and glucose uptake. Mol Cancer Ther 7: 2599-2608.

S20. Zhang M-Y, Feng Y, Wang Y, Dimitrov DS (2009) Characterization of a monoclonal antibody against the insulin-like growth factor-I receptor. mAbs 1: 475-480.
