# Supplementary material for: Evaluation of a Novel Hexavalent Humanized Anti-IGF-1R Antibody and Its Bivalent Parental IgG in Diverse Cancer Cell Lines
Source: PLoS One. 2012 Aug 31;7(8):e44235. doi: 10.1371/journal.pone.0044235 (PMC3432068; doi:10.1371/journal.pone.0044235)
Supplement: Table S2 — N-terminal protein sequencing of R1. (DOC) [file pone.0044235.s011.doc]

**Table S2. N-terminal protein sequencing of R1**

| Cycle/position | 1 | 2 | 3 | 4 | 5 | 6 | 7 | 8 | 9 | 10 | 11 | 12 | 13 | 14 | 15 |
| --- | --- | --- | --- | --- | --- | --- | --- | --- | --- | --- | --- | --- | --- | --- | --- |
| (H + L)a | D/E | I/V | K/V | L/M | T/V | E/Q | S | G/H | G/K | F/G | L/M | S/V | Q/T | P/S | G/V |
| VHb | E | V | K | L | V | E | S | G | G | G | L | V | Q | P | G |
| VKb | D | I | V | M | T | Q | S | H | K | F | M | S | T | S | V |

^a^Purified R1 was subjected to N-terminal protein sequencing (15 cycles). Two residues were detected after each cycle of Edman degradation.

^b^Deduced from the DNA sequences.
