# Supplementary material for: Evaluation of a Novel Hexavalent Humanized Anti-IGF-1R Antibody and Its Bivalent Parental IgG in Diverse Cancer Cell Lines
Source: PLoS One. 2012 Aug 31;7(8):e44235. doi: 10.1371/journal.pone.0044235 (PMC3432068; doi:10.1371/journal.pone.0044235)
Supplement: Table S3 — Binding of 125I-IGF-1 to MCF-7L in the presence of MAB391 or R1. (DOC) [file pone.0044235.s012.doc]

Table S3. Binding of 125I-IGF-1 to MCF7-L in the presence of MAB391 or R1

| [Ab] | MAB391 | R1 |
| --- | --- | --- |
| 1000 ng/mL | 38 % | 58 % |
| 100 ng/mL | 54 % | 71 % |
| 10 ng/mL | 95 % | 97 % |
| 0 ng/mL | 100 % | 100 % |
